# SARS-CoV-2 Membrane Protein: From Genomic Data to Structural New Insights

**DOI:** 10.3390/ijms23062986

**Published:** 2022-03-10

**Authors:** Catarina Marques-Pereira, Manuel N. Pires, Raquel P. Gouveia, Nádia N. Pereira, Ana B. Caniceiro, Nícia Rosário-Ferreira, Irina S. Moreira

**Affiliations:** 1 CNC—Center for Neuroscience and Cell Biology, University of Coimbra, 3004-535 Coimbra, Portugal; catarina.103@gmail.com (C.M.-P.); manuelmoreirapires@hotmail.com (M.N.P.); raquelpinagouveia@gmail.com (R.P.G.); nadia.pereira.nnp@gmail.com (N.N.P.); a.beatrizcaniceiro@gmail.com (A.B.C.); nirferreira@hotmail.com (N.R.-F.); 2 IIIs—Institute for Interdisciplinary Research, University of Coimbra, 3030-789 Coimbra, Portugal; 3 Department of Sciences, University of Porto, 4169-007 Porto, Portugal; 4 CQC—Coimbra Chemistry Center, Chemistry Department, Faculty of Science and Technology, University of Coimbra, 3004-535 Coimbra, Portugal; 5 Department of Life Sciences, University of Coimbra, Calçada Martim de Freitas, 3000-456 Coimbra, Portugal; 6 Center for Innovative Biomedicine and Biotechnology, University of Coimbra, 3004-535 Coimbra, Portugal

**Keywords:** SARS-CoV-2, genomics, proteomics

## Abstract

Severe Acute Respiratory Syndrome CoronaVirus-2 (SARS-CoV-2) is composed of four structural proteins and several accessory non-structural proteins. SARS-CoV-2’s most abundant structural protein, Membrane (M) protein, has a pivotal role both during viral infection cycle and host interferon antagonism. This is a highly conserved viral protein, thus an interesting and suitable target for drug discovery. In this paper, we explain the structural nature of M protein homodimer. To do so, we developed and applied a detailed and robust in silico workflow to predict M protein dimeric structure, membrane orientation, and interface characterization. Single Nucleotide Polymorphisms (SNPs) in M protein were retrieved from over 1.2 M SARS-CoV-2 genomes and proteins from the Global Initiative on Sharing All Influenza Data (GISAID) database, 91 of which were located at the predicted dimer interface. Among those, we identified SNPs in Variants of Concern (VOC) and Variants of Interest (VOI). Binding free energy differences were evaluated for dimer interfacial SNPs to infer mutant protein stabilities. A few high-prevalent mutated residues were found to be especially relevant in VOC and VOI. This realization may be a game-changer to structure-driven formulation of new therapeutics for SARS-CoV-2.

## 1. Introduction

COronaVIrus Disease 2019 (COVID-19) is currently a worldwide pandemic that was first reported in December 2019 in Wuhan, China, and, since then, led to more than 446 M infected people and over 6.00 M deaths [1] (as of 7 March 2022). COVID-19 is caused by Severe Acute Respiratory Syndrome CoronaVirus-2 (SARS-CoV-2), which is a Coronaviridae family, positive single-stranded RiboNucleic Acid (ssRNA) virus [2,3]. Since the beginning of this pandemic, SARS-CoV-2 has mutated over time, leading to the identification of several variants that, based on phylogeny [4], have been organized into clades named L, S, V, G, GH, GR, GV, GRY, and O (clade based on exclusion encompassing sequences that do not fit into other clades) [5,6].

According to the World Health Organization (WHO), there are Variants Of Interest (VOI), variants that have been recognized as being able to acquire community transmission causing clusters and being further identified in several countries, or assessed as a VOI by WHO’s SARS-CoV-2 Virus Evolution Group. On the other hand, Variants Of Concern (VOC) are variants that, adding to the characterization as VOI, are linked to increased transmissibility or virulence, and/or a decrease in the effectiveness of treatment, prevention, and diagnosis approaches currently used. VOI are distributed among clades G (lineages B.1.525 and B.1.617.1), GH (lineages B.1.427/B.1.429 and B.1.526), and GR (lineages C.37, P.2, and P.3). Moreover, VOC are distributed among clades G (lineage B.1.617.2), GH (lineage B.1.351), GR (lineage P.1), and GRY (lineage B.1.1.7).

SARS-CoV-2 genes encode four major structural proteins: Spike (S) protein, Membrane (M) protein, Nucleocapsid (N) protein, and Envelope (E) protein. Along with these structural proteins, SARS-CoV-2 genes also encode sixteen non-structural proteins (nsp) and accessory proteins [7]. One of the most conserved structural proteins in SARS-CoV-2 is the M protein, as it has a smaller mutation rate sharing structural and functional similarities with M proteins from another coronavirus [8]. M protein is also the most abundant structural protein playing a central role in directing virus assembly and budding via interaction with structural proteins E, S, and N through heterotypic interactions [9,10,11]. In addition to these heterotypic interactions, M protein is functionally dynamic and can also acquire a homodimeric form [9,12,13]. Haan et al. demonstrated the existence of M-M interactions, using co-immunoprecipitation and envelope incorporation assays, and determined the M protein domains involved in this homotypic association, using a mutagenesis approach [12]. Neuman et al. demonstrated that M−M interactions may take various forms, using cryo-Electron Microscopy (cryo-EM) in structural M protein models of SARS-CoV [9]. Yu et al. simulated M–M interactions using Coarse-Grained Molecular Dynamics simulations (CGMD) [14]. Ouzounis et al. used the Orf-3a as a structural template to predict low-resolution three-dimensional models of M proteins [15]. Interactions between M and N proteins stabilize virion RNA genome [9,10,11,16], making this Protein–Protein Interaction (PPI) a potential drug target [17]. Interactions between M protein and E proteins induce membrane curvature, strong enough to assemble and release virus-like particles [9,10,11,16]. Interactions between M and S proteins are essential for S protein retention in Endoplasmic Reticulum–Golgi Intermediate Compartment (ERGIC) and its integration into new virions [16]. Overall, M protein interferes with host immunological response through interferon antagonism, is involved in host cell cycle arrest, induces Endoplasmic Reticulum (ER) stress and unfolded protein response, has a role on coronavirus-induced autophagy, and has a protective antigen function [18]. In addition, M protein homodimers formation is essential for coronavirus envelope assembly [9,12,19,20]. Since M protein is essential in the SARS-CoV-2, including for Virus-like particles (VLP) formation, a complete understanding of the structure–function relationship can help the development of more efficient therapeutics [9,19,21]. However, this task has been affected by the difficulty to stabilize and crystallize the M protein [22,23,24], and as such there is no experimentally resolved structure available by either Nuclear Magnetic Resonance (NMR), X-ray crystallography, or cryo-EM [25], thus far.

M protein is constituted by 223 amino acids and has three major domains: a short- N-terminal ecto-domain, three TransMembrane Helices (labelled as TMH1, TMH2, and TMH3), and a long C-terminal endo-domain located on the cytoplasmic face of virions [12,13,19,26]. There are few reports of M protein homodimers, but those that exist explain only in part the process of homodimer formation through M–M interactions [9,12,13,14,15,27]. M protein molecules interact with each other through multiple contact sites, especially in transmembrane domains [12]. An experiment on SARS-CoV M protein (which shares 90.5% sequence identity with SARS-CoV-2 M protein [28]) demonstrated that residues W19, W57, P58 W91, Y94, F95, and C158 play a key role in homodimer interactions, suggesting that homologous residues W20 (TMH1 domain), W58, P59 (TMH2 domain), W92, Y95, and F96 (TMH3 domain) of SARS-CoV-2 may be important for M dimer interaction and stabilization [13]. Moreover, SARS-CoV cysteine residues C63, C85, and C158 mutations did not interfere with M dimer formation, suggesting that homologous SARS-CoV-2 M protein residues C64, C86, and C159 may also not be involved in M dimer interface [13].

**Figure 1:**
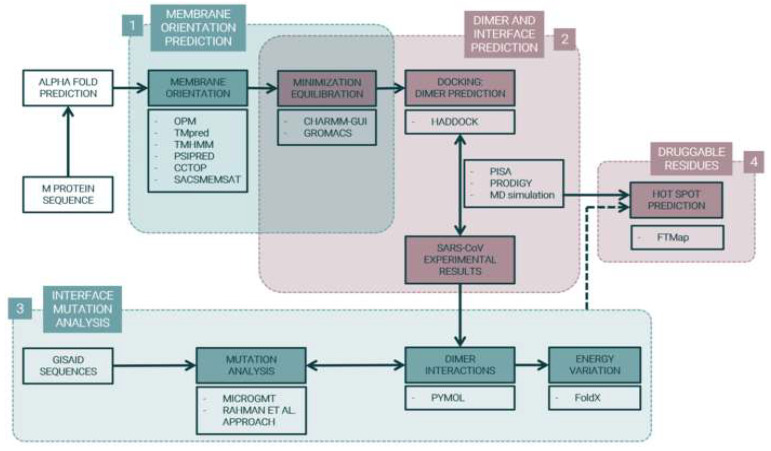
Project Pipeline. M protein structure was predicted by AlphaFold [31]. (1) Membrane orientation was predicted with Orientations of Proteins in Membranes (OPM) [32], prediction of Transmembrane Helices (TMpred) [33], TransMembrane prediction using cyclic Hidden Markov Model (TMHMM) [34,35], Prediction of secondary structure (PSIPRED) [36,37], Consensus Constrained TOPology prediction (CCTOP) [38,39], and Sequence Analysis and Consulting Service MEMbrane protein Structure And Topology (SACSMEMSAT) [40]. Protein–membrane systems were constructed with Chemistry at HARvard Macromolecular Mechanics Graphical User Interface (CHARMM-GUI) [41] and minimization and equilibration were conducted using GROningen MAchine for Chemical Simulations (GROMACS) [42,43]. (2) M protein dimer was predicted with High Ambiguity Driven protein–protein DOCKing (HADDOCK) [44] and results were compared to SARS-CoV experimental data. (3) Gene and protein mutations were analyzed with Microbial Genomics Mutation Tracker (MicroGMT) [45] and Rahman et al. [46] programs and energy variation of mutations in dimer interaction residues were calculated with FoldX [47]. (4) Druggable residues in SARS-CoV-2 Membrane protein dimer were predicted through FTMap [48] hotspot clusters.

Bioinformatic tools are well established methodologies that allow to attain a structural and functional characterization of relevant biomedical targets for viral infections and to predict new virus-host interactions [29,30]. In this work, through an in-house developed in silico approach (Figure 1), we modelled the M protein monomer and dimer three-dimensional (3D)-structures along with predictions for their membrane orientation and homodimeric interface. We also predicted the impact of mutations in the predicted homodimeric interface, and the best region for the binding of new drugs/peptides, paving the way to structure-driven formulation of new therapeutic solutions.

## 2. Results

Due to the lack of an M-protein experimental structure, we have used the AlphaFold predicted structure and subjected it to a MD simulation protocol. AlphaFold [31] algorithm was shown to predict protein structures, attaining experimental resolution, as already demonstrated in the challenging 14th Critical Assessment of protein Structure Prediction (CASP14). Furthermore, Leo et al. [49] showed the potentiality of using 3D structures of proteins attained by machine learning algorithms, and further refined them by a physics-based protocol, as followed in our study.

### 2.1. M Protein Monomer Structure and Membrane Orientation

M protein is a membrane protein and the determination of its correct orientation in the lipid bilayer membrane is needed to understand its main interactions, and, therefore, its biological function. To this end, six different web-based resources for membrane orientation prediction were used: OPM [32], TMpred [33], TMHMM [34,35], PSIPRED [36,37], CCTOP [38,39], and SACSMEMSAT [40].

M protein Root-Mean-Square-Deviation (RMSD) results were obtained considering residues from the whole protein (monomer RMSD), TMH1, TMH2, C-terminal, and N-terminal, using the AlphaFold monomer structure as reference. M protein monomer predicted residue domains, after system equilibration by Molecular Dynamic (MD) simulations, were very similar for all membrane orientation predictions. Monomer RMSD values were 1.61 ± 0.26 Å for TMHMM, 1.38 ± 0.18 Å for CCTOP, 1.36 ± 0.22 Å for TMpred, 2.38 ± 0.59 Å for SACSMEMSAT, 1.83 ± 0.35 Å for OPM, and 2.95 ± 0.28 Å for PSIPRED predictions (Supplementary Figures S1 and S2). For the following dimer prediction study, PSIPRED results were not used as RMSD values were higher for both monomer and transmembrane helices 1 and 2 RMSDs. Despite SACSMEMSAT and CCTOP having comparable values to the other predictors, they showed an arched TMH1 after an equilibration MD simulation that could influence dimer stability (Supplementary Figure S2). Hence, out of the six membrane predictors used initially, OPM, TMHMM, and TMpred M protein monomers were chosen for further analysis.

**Figure 2:**
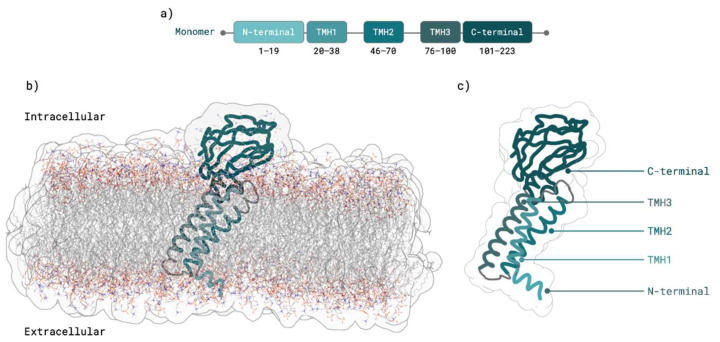
SARS-CoV-2 M protein monomer. (**a**) M protein domains predicted by TMHMM [34,35] membrane predictor. (**b**) TMHMM [34,35] M protein monomer structure prediction after equilibration in membrane with ER membrane composition. (**c**) M protein structure with domains highlighted.

OPM, TMpred, and TMHMM M monomers from the previous step were used to model dimer 3D structures using a well-established protein–protein docking software: HADDOCK [44]. From 3000 proposed docking decoys, 1000 for each membrane orientation, 20 dimer structures that respected the membrane orientation prediction were selected: 11 from OPM, 4 from TMpred, and 5 from TMHMM. From these 20 dimers, two structures from the TMHMM membrane predictor were chosen based on their similarity with SARS-CoV experimental detected interactions, namely in TMH2 (P59) and TMH3 (W92, L93, F96) regions [13]. From these two TMHMM M protein dimers, the final choice was based on PROtein binDIng enerGY (PRODIGY)’s metrics of biological probability and predicted binding affinity. Hence, the M protein dimer structure chosen for the proceeding studies showed 85.6% biological probability and a predicted binding affinity of −6.3 kcal/mol in comparison to 74.8% biological probability and −5.9 kcal/mol binding affinity results from the other chosen structure. Regarding the TMHMM monomer membrane prediction that served as template for the final chosen dimer, M protein monomer residues 11–19 were shown to stably belong to N-terminal domain, residues 100–203 to C-terminal domain, residues 20–38 to TMH1, residues 46–70 to TMH2, and residues 76–100 to TMH3 (Figure 2).

### 2.2. M Protein Dimer and Interface Prediction

#### 2.2.1. Dimer Prediction

**Figure 3:**
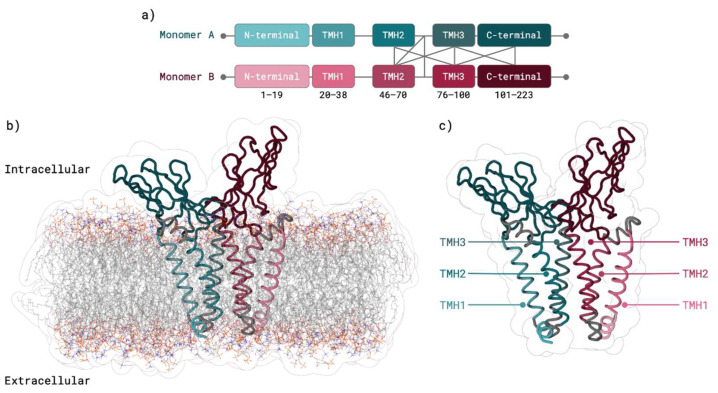
SARS-CoV-2 M protein dimer HADDOCK [44] prediction using TMHMM [34,35] based monomers. (**a**) Interaction representation between Monomer A (teal), and Monomer B (garnet) domains. (**b**) M protein dimer within the membrane: Monomer A (teal), and Monomer B (garnet). (**c**) M protein dimer with TMH domains highlighted: Monomer A (teal), and Monomer B (garnet).

The final dimer 3D structure (Figure 3) was subjected to three independent dimer system MD replicas of 0.5 μs. Minimum distance, radius of gyration and RMSD results in function of MD simulation time are further described in Supplementary Figures S3–S5, respectively. After equilibration, polar contacts between M protein monomer and membrane lipids occurred in M monomer residues K14, Y39, R42, N43, R44, F45, Y71, R72, W75, S94, R101, R107, W110, S173, and R174. Transmembrane regions were within membrane lipids throughout the entire equilibration and several M protein residues were able to establish polar contacts with membrane lipids, supporting our transmembrane region assessment (Figure 3).

RMSD results (Supplementary Figure S6) showed that monomer A and monomer B behaved differently throughout the MD simulation. In monomer A, TMH3 domain was the most stable region. Monomer A TMH2 domain interacted with monomer B and was a bit more unstable when compared with TMH1 domain (Supplementary Figure S6a). In monomer B, TMH domains were also very stable, presenting however a much higher deviation and lower stability of the N- and C-terminus compared with other domains (Supplementary Figure S6b). Root-Mean-Square-Fluctuation (RMSF) results (Supplementary Figure S7) for monomer A and monomer B were very similar. As expected, TMH residues, in large majority α-helices, showed low fluctuation, whilst C-terminus residues, present in a random coil, presented higher fluctuation. Cross-Correlation Analysis (CCA) results (Supplementary Figure S8) showed that within both monomers, TMH2 is highly positively correlated (moves in the same direction) with TMH1 and TMH3 within the same protein. On the contrary, between monomers, TMH1 and TMH2 showed a negative correlation (moving in opposite directions) with remaining helices of the opposite monomer. Average ΔSolvent accessible surface area (SASA) for the interfacial residues only showed small variations, further strengthening the stability of the established homodimer interface (Supplementary Figure S9).

#### 2.2.2. Interface Analysis

**Figure 4:**
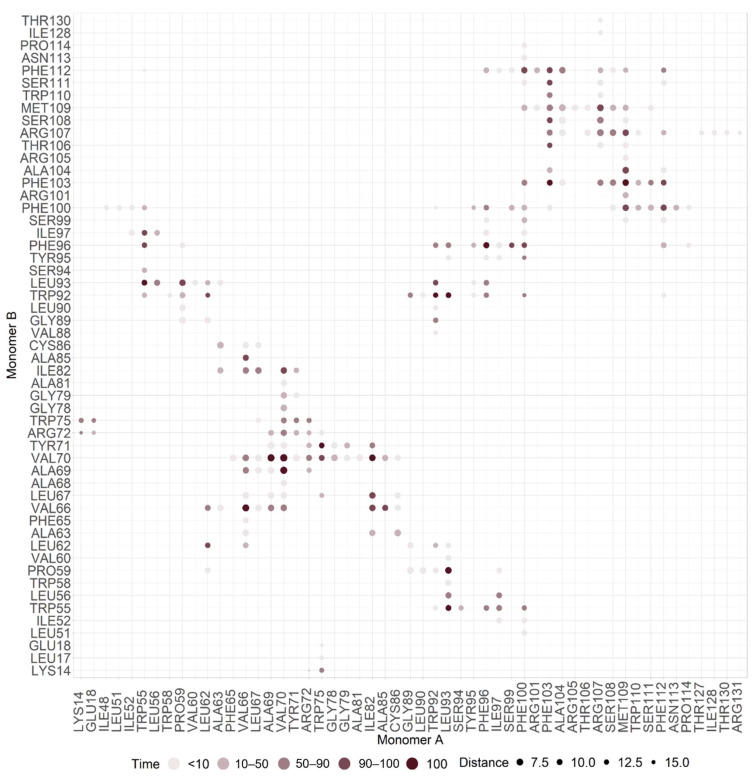
Contact map of M protein homodimer interface.

After dimer equilibration in an ER membrane used to mimic the expected biological environment, we showed that the dimer interface was composed of 38 residues, 17 from monomer A (W55, P59, L62, V66, A69, V70, W75, I82, A85, W92, L93, F96, F100, F103, R107, M109, and F112) and 21 residues from monomer B (P59, L62, V66, A69, V70, Y71, I82, A85, W92, L93, F96, I97, F100, F103, A104, R107, S108, M109, S111, and F112). These residues established 34 pairwise interactions, showing high proximity and high prevalence time (90% cut-off) (Table 1). Carbon Alpha (Cα) distances of interacting residues varied between 5.25 Å (V70–V70 residues interaction) and 12.58 Å (W92–W92 residues interaction), with a mean Cα distance of 9.57 ± 0.60 Å. From these residues, 12 (P59, V66, A69, V70, I82, L93, F96, F100, F103, R107, M109, and F112) interacted in both monomers. From these 38 residues, 23 were unique residues, seven from TMH2 (W55, P59, L62, L67, V66, A69, and V70), two from TMH2-TMH3 extracellular loop (Y71, and W75), seven from TMH3 (I82, W92, L93, I97, A85, F96, and F100) and seven from C-terminal (F103, A104, R107, S108, M109, S111, and F112) (Table 1). From these, 8 were aromatic (Y71, W55, W75, W92, F96, F100, F103, and F112), 20 non-polar (W55, P59, L62, V66, L67, A69, V70, Y71, W75, I82, A85, W92, L93, F96, I97, A104, F100, F103, M109, and F112), 3 polar (S108, S111, and R107) and 1 was a positively charged residue (R107). Figure 4 provides the contact map of this dimer interface.

**Figure 5:**
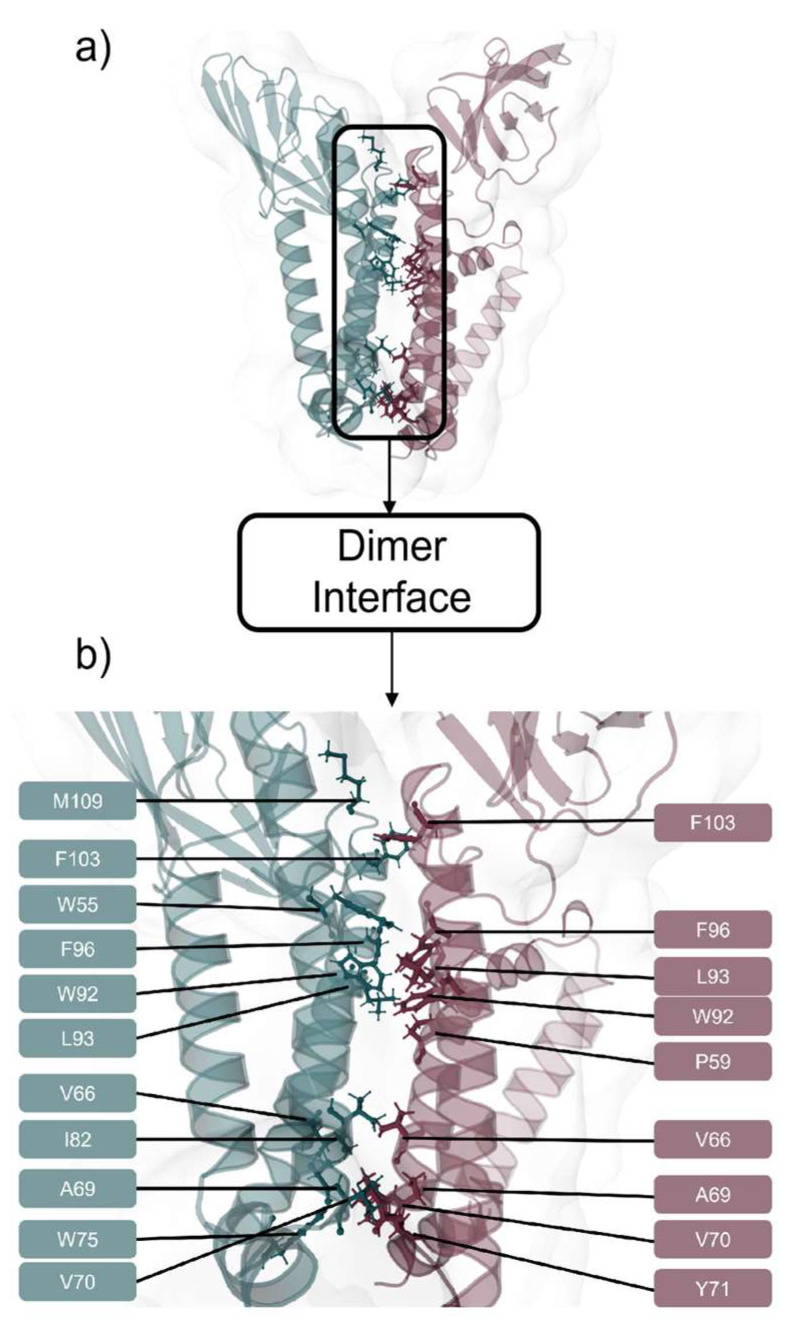
(**a**) SARS-CoV-2 M protein dimer via HADDOCK [44] prediction using TMHMM [34,35] based monomers with interfacial residues represented as sticks, and (**b**) interface zoom-in featuring interfacial residues identified with the color code of teal for Monomer A and garnet for Monomer B.

Interactions between monomer A and monomer B residues W59-L93, V66-V66, A69-V70, V70-A69, V70-V70, W75-Y71, I82-V70, W92-W92, L93-P59, F96-F96, F103-F103, and M109-F103 were prevalent interactions throughout 100% of MDs simulation time, with side chain distances lower than 5 Å (Table 1, Figure 4 and Figure 5). These regions also showed a low fluctuation (e.g., low RMSF values). Hydrophilic interactions occurred between monomer A residues L62-V66, V66-V69, W92-F96, F96-F100, and F103-R107 and between monomer B residues L62-V66, V66-V69, L92-I97, F100-A104, A104-R107, S106-M107, and M107-F112. π-π stack interactions occurred between monomer A residues W92-F96 and F100-F112 and between monomer A and monomer B residues W55-F100, W92-W92, F100-F112, and F103-F103, respectively.

### 2.3. M Protein Mutation Analysis

**Figure 6:**
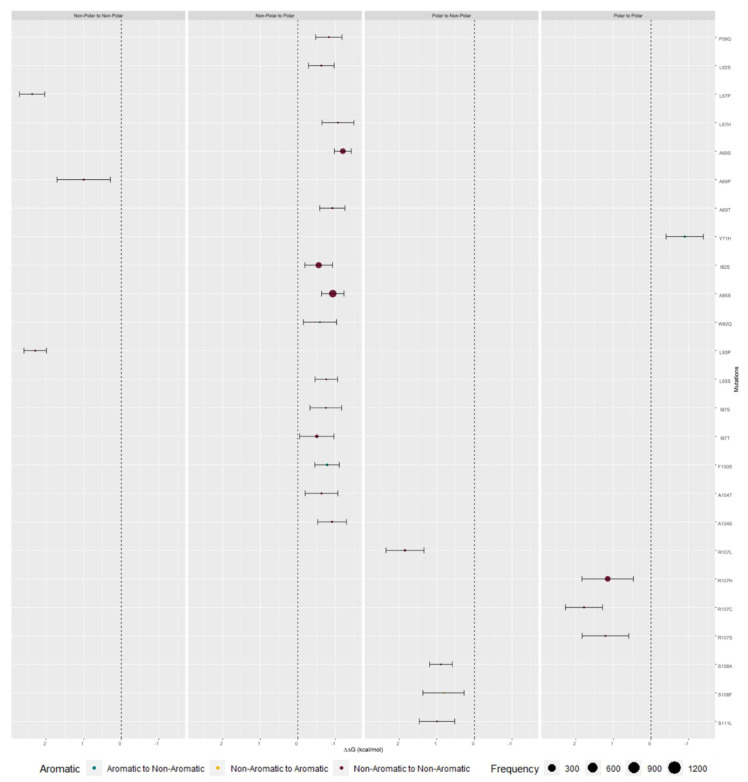
ΔΔG_binding_ values of predicted interfacial residues that possessed mutations capable of causing major impact in protein stability. Color represents the alteration from aromatic to non-aromatic (teal), non-aromatic to aromatic (yellow), and non-aromatic to non-aromatic (garnet) (all the presented results are mean values ± standard deviation).

We retrieved 1271550 M protein sequences, submitted between 10 January 2020 and 3 May 2021 from 180 countries, from the Global Initiative on Sharing All Influenza Data (GISAID) [50,51] database. Genomic sequences were obtained from human hosts, with more than 29,000 bases per sequence, and less than 5% missing values. The sequence distribution retrieved across GISAID clades and across the world can be observed in Supplementary Figure S9. Clades S, G, GH, and GR encompass sequences that are most prevalent in North America. The latter clade is also well represented in the Oceania region. Clades GV and GRY are most prevalent in Europe and clades O and L are sparse across the world (Supplementary Figure S10). Within the M protein interfacial residues from analyzed sequences, 91 Single Nucleotide Polymorphisms (SNPs) were retrieved from 21868 sequences. FoldX was used to assess the binding free energy differences between mutated and Wild-Type (WT) proteins ΔΔG_binding_) and the respective values by physico–chemical character of the analyzed mutation are illustrated in Figure 6 and with higher detail in Supplementary Figure S11.

#### 2.3.1. Single Mutation Analysis

To assess the mutation effect at the dimer complex, we have focused on the reported mutations at the predicted interface. In these considered regions the vast majority of the mutations did not impact protein stability in a significant manner (Supplementary Figure S11, Table 2). Most mutations were found in non-polar residues and did not significantly impact protein stability. As expected, changes in polarity, which could have higher consequences in the microenvironment around them, led to more significant alterations of dimer stability. This was particularly true when changing from a polar to a non-polar residue (Supplementary Figure S11, Table 3). Aromaticity changes also seem key in this dimer interface as mutations from non-aromatic residues towards aromatic represent the predominant change. None of the possible aromatic changes led to a significant change in protein stability. (Supplementary Figure S11, Table 4).

SNP I82T, located at the TMH3 domain, was the most common SNP detected. This mutation led to the residue’s polarity modification from a non-polar residue into a polar one and occurred in 6316 (28.88%) sequences from our dataset. The second most frequent SNP was V70L, at the end of the TMH2 domain. This mutation did not change the type of polarity at that specific position and was detected in 6303 (28.82%) sequences. Physico–chemical properties of valine and leucine are similar, and V70L mutation was shown to be inconsequential for dimer stability. However, this is not true for isoleucine and threonine, two amino acids with different polarity, and I82T leads to a stability gain. Interestingly, these two residues, I82 and V70, are interacting strongly throughout the entire MDs simulation (Table 1, Figure 5). These were by far the most common SNPs, with the third most common one occurring in only 1455 sequences (more details in Supplementary Table S1).

#### 2.3.2. Mutation Distribution in Variants

We also analyzed the type of mutation found in each known clade (Supplementary Figure S11, Supplementary Table S1—single mutations and Table S2—co-occurring mutations). The most common mutated clade was GRY, and the most frequent mutation found in this clade was V70L (Table 5). This mutation co-occurred in GRY with M109L (8 cases), A104V (2 cases), and A69F (1 case) without any major identifiable energetic advantage (ΔΔG_binding_ around 0 kcal/mol) (Supplementary Table S2). The second most frequent mutated clade, where VOCs are also located, was GH and the most frequent mutation in this clade was I82T (Table 5). A few mutations also co-occurred with I82T but in low frequency. From these, A85S induced a higher stabilization of the dimer interface (ΔΔG_binding_ value of −1.47 ± 0.47 kcal/mol) (Supplementary Table S2). G clade was the third most mutated clade, and the most frequent one was I82T (Table 5). A few double mutations of interfacial residues were also found, in particular I82T-R107L (4 cases), I82T-V70F (2 cases), I82T-M109I (2 cases), I82T-V66M (2 cases), I82T-A85S (2 cases), and I82T-R107H (2 cases) but none led to higher changes in the binding free energy (Supplementary Table S2). GR clade was the following most mutated and the most common mutation in this clade was V70F (Table 5). A few mutations were found in association, such as A85S (3 cases, ΔΔG_binding_ = −0.72 ± 0.64 kcal/mol) and A104V (1 case, ΔΔG_binding_ = 0.10 ± 0.54 kcal/mol) (Supplementary Table S2). The remaining clades were much less populated with mutated sequences.

**Figure 7:**
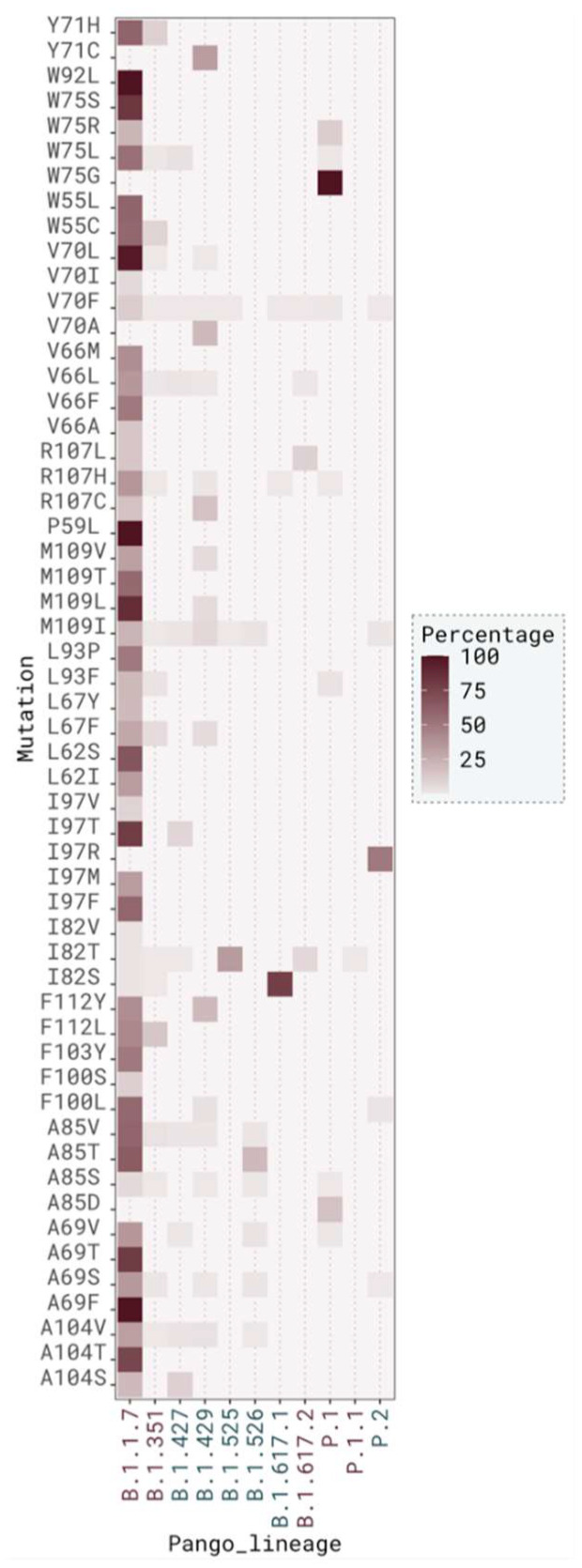
Distribution across VOC (garnet) and VOI (teal) of SARS-CoV-2 M protein sequences.

In total, there were 8951 (40.93%) mutated sequences that were found in VOC and 2757 (12.61%) that were found in VOI. Out of VOC identified sequences, 8474 (94.67%) were contained in pango lineage B.1.1.7 and the most common mutation in this variant was V70L, represented in 6136 sequences (72.41%). In sequences identified as VOI, the most represented pango lineage was B.1.525 (72.59%) and the most frequent mutation for this variant was I82T, present in 2139 sequences (72.48%) (Figure 7 and Supplementary Figure S12).

#### 2.3.3. Druggable Hot Spots

Solvent occlusion has already been demonstrated as a key aspect of PPIs, as main interfacial residues SASA values are considerably more diminished upon complex formation compared to other interfacial residues [52,53,54,55,56,57]. Two metrics are particularly relevant, ΔSASA and relSASA, as the first one allows us to quantify occlusion upon complex formation and the second, which represents the quotient between ΔSASA and SASA_monomer_, allows us to distinguish between residues with the same ΔSASA but with different solvent accessibility in the monomer. The most mutated residues such as I82, V70, and A69 showed higher ΔSASA and relSASA values, which indicates occlusion of these residues upon complex formation, with SASA_complex_ values tending to zero (higher ΔSASA and _rel_SASA closer to 1) (Table 6, Supplementary Table S1). Other frequently mutated residues lose accessibility to the solvent but remain attainable in the complex form: e.g., M109, A104, R107, and W75. By preventing bulk water to approximate these interfacial residues, the number and force of interaction established increases and the PPI is strengthened. Residues V70, M109, and I82 established a high number of dimer interactions: 6, 5, and 4, respectively. On the other hand, residues A69, R107, and W75 established two interactions each and residue A104 established only one interaction (Table 6, Supplementary Table S1).

As some of these mutations may impact protein’s stability, we also investigate the identification of their presence in VOI and VOC key strains since it can lead to future drug discovery concerning the M protein. The mutations leading to ΔΔG_binding_ below −0.50 kcal/mol or over 0.50 kcal/mol are indicative of such cases. Mutations A69P, R107C, R107H, R107L, and R107S, all have ΔΔG_binding_ values over 0.50 kcal/mol. Despite the R107H relatively low mutation frequency, it appears in several VOCs as B1.1.7, B.1.351, P.1, and VOI B.1.617.1. On the other hand, mutations I82T, I82S, A69S, A104S, A69T, and A104T have ΔΔG_binding_ values below −0.50 kcal/mol meaning that they have a favorable impact on the mutated protein stability. Mutation I82T has been detected in several VOCs as B.1.617.2 and B.1.1.7, in higher frequency, but also in P.1.1 and B.1.351, and in VOI B.1.525. Mutation I82S has been detected in VOCs B1.1.7 and B.1.351 sparingly and in VOI B.1.617.1 more frequently. Mutation A69S has been detected in VOC B.1.1.7 more frequently than in VOC B.1.351 and in VOI B.1.526 much more infrequently, and in VOI P.2 just once. Mutation A69T is much less frequent than A69S but has also been detected in VOC B.1.1.7. Finally, mutations A104S and A104T have both been identified in VOC B.1.1.7 twice and three times, respectively.

**Figure 8:**
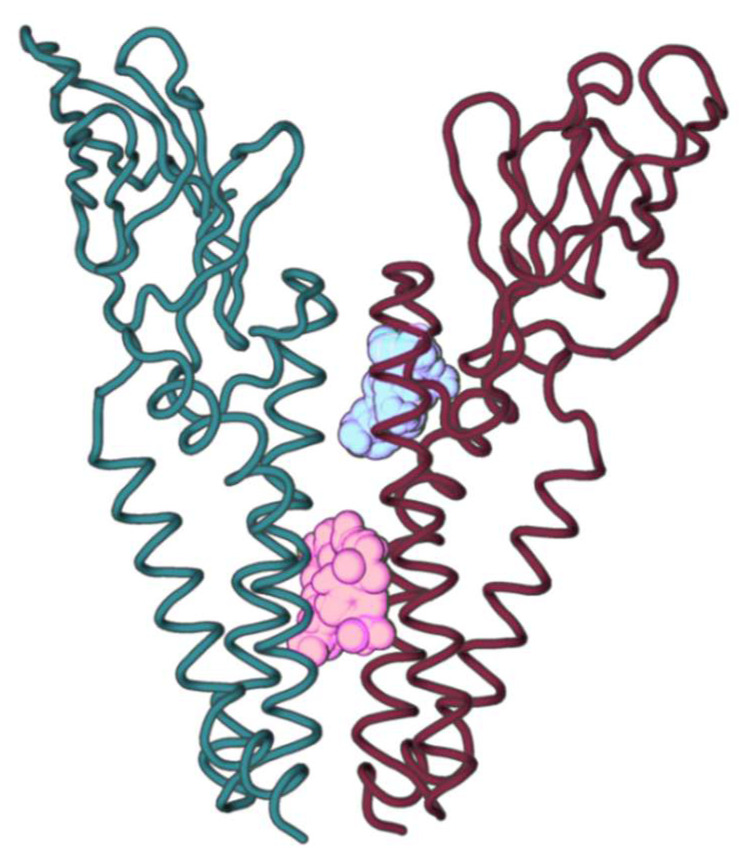
SARS-CoV-2 M protein homodimer and the two FTMap [48,58,59,60] clusters identified within the interface region. The highest-ranking cluster is represented in teal and the second highest cluster is represented in garnet.

In order to understand if any of the previously mentioned mutation points were druggable residues, the production MD phase was clustered and subjected to FTMap [48,58,59,60], to further characterize potential ligand hotspot positions for new drugs. FTMap [48,58,59,60] probes were shown to cluster into two different positions: one on the transmembrane zone near to C-terminal (teal) and another between TMH2 and TMH3 (garnet), deeper into the membrane (Figure 8). The teal cluster displayed the highest FTMap [48,58,59,60] score and the attained probes interacted with M protein dimer predicted interface residues F96, I97, F100, and F103 in Monomer A and with residues W55, S108, M109, S111, and F112 in Monomer B. The bulk of mutations for the residues that interact with this probe are mutations that don’t impact protein stability or have a positive effect on stability (Table 7). Probes from this cluster also interacted with Monomer B residues Y47, L51, I52, Y95, W110, N113, P114, and G115, but these are not predicted homodimer interacting residues.

In the second cluster (garnet), probes interacted with predicted M protein dimer interface residues P59, V66, W92, L62, L67, A85, and L93 from Monomer A and B and I82 from Monomer B. Similarly, to the highest scoring cluster, the mutations on residues that interact with the second cluster are also mainly not impactful on protein stability or impact it in a positive manner. Even though some mutations are more frequent in these residues, all in all they are still very conserved (Table 8). Probes from this cluster also interacted with residues V60, A63, A81, A83, C86, G89, L90, M91, and S94 from Monomer A and V60, A63, C86, G89, and L90 from Monomer B, but these are not predicted homodimer interacting residues. Other clusters from FTMap [43,53,54,55] were not considered as their probes were not interacting in the predicted interface region.

Overall, the highest score cluster interacted with very conserved interfacial residues whereas the second highest cluster was in a region constituted by residues more prone to stabilizing mutations (especially mutations that alter the chemical character of the interface).

## 3. Discussion

### 3.1. M Protein Monomer Structure and Membrane Orientation

At the moment, a few predictions for the M protein monomer were made as is the case for Heo and Feig [49], Zhang et al. [61], and AlphaFold [62]. In this work our starting point 3D model was the one developed by a state-of-the-art methodology, AlphaFold’s, which was also determined as adequate and in consensus with other predictions [49]. We predicted its membrane orientation using six different membrane orientation softwares. After minimization and MD equilibration, we chose TMHMM M protein monomer membrane orientation prediction for the following studies since it showed a higher stability, with low RMSD values upon comparison with the initial AlphaFold’s structure, and without any major conformational change. SARS-CoV M protein monomer domains were previously predicted in experimental research that elucidated M protein dimer interactions [13]. In that experiment, residues 15-37 were shown to belong to TMH1, residues 50–72 to TMH2 and residues 77–99 to TMH3 [13]. Herein, for the first time, a detailed SARS-CoV-2 M protein membrane orientation was proposed, showing that residues 20–38 belong to TMH1, residues 46–70 to TMH2, and residues 76–100 to TMH3, results in agreement to the above-mentioned SARS-CoV experimental results. We also show a comparison between our equilibrated model structure against two other relevant predicted structures (Supplementary Figure S13). The overall conformation is similar, and the highest differences were found at TMH2 and TMH1, in particular in the length and linearity of TMH1. Supplementary Figures S2 and S6 clearly show that these regions are very stable through the MD simulation, further strengthening our chosen model 3D structure.

### 3.2. M Protein Dimer and Interface Prediction

Despite the M protein dimer being crucial for various biological functions such as SARS-CoV-2 virion assembly and shape formation [63], the type of interactions established in its homodimer form are still poorly understood [9,12]. Experimental SARS-CoV M protein dimer data demonstrated that residues W19, W57, P58, W91, L92, Y94, F95, and C158 were relevant, suggesting that homologous residues W20 (TMH1 domain), W58, and P59 (TMH2 domain), and W92, L93 Y95, and F96 (TMH3 domain) of SARS-CoV-2 may also be important for M dimer interaction and stabilization [13]. Authors also hypothesized that SARS-CoV residues C63, C85, and C158 mutations did not interfere with M dimer formation, suggesting that homologous SARS-CoV-2 M protein residues C64, C86, and C159 may also not be involved in M dimer interface [13]. A previous in silico approach proposed a M protein dimer structure based on four different templates (PDB IDs: 3A7K_A [64], 5UTT_A [65], 6SPB_V [66], and 6XDC [67]), and authors hypothesized the interaction was established between TMH1 and TMH2 [68]. These results differ from the experimental results on SARS-CoV that showed dimer interactions between monomers TMH2 and TMH3, as well as from other in silico studies suggesting that these regions interact [69]. However, other authors also attained agreement with experimental results with SARS-CoV dimer interactions [69]. Herein, SARS-CoV experimental information was used as cue for various docking experiments as already detailed in the Results section. A high confidence docking decoy based on the TMHMM monomer was subjected to further studies due to its proper membrane orientation regarding previous analysis. It was subjected to 1.5 μs MD, which showed that overall conformational stability for monomer A and monomer B was slightly different (e.g., dissimilar RMSDs), whereas RMSF results were alike, especially in TMH domains. TMHs showed low fluctuations, which allowed the establishment of highly prevalent and meaningful interactions between the two monomers. We identified 34 main interactions responsible for the M protein dimer 3D structure stabilization, between 17 residues from monomer A and 21 residues from monomer B. From these interactions, 73.53% occurred between transmembrane residues, which was expected as the M protein is a transmembrane dimeric system. From these interactions, 12 were conserved throughout the entire MD simulation time, including interactions between W92-W92, L93-P59, and F96-F96, homologous residues from the ones detected to SARS-CoV [13]. This suggests that these three interactions are pivotal towards M protein dimer stabilization. Other interacting residues were present in lasting interactions throughout the MDs simulations, and thus important residues to further study and validate were W55, V66, A69, V70, Y71, W75, I82, L93, F103, and M109.

Feig’s laboratory has also proposed two possible configuration arrangements for the M protein homodimer [70], which were named by Monje-Galvan et al. as “open” and “closed” conformations [69]. The dimer structure configuration obtained in our study is similar to the “open” one (Supplementary Figure S14), and it is also supported by other authors, such as Cao et al. [27]. Monje-Galvan et al. [69] also described the interface region of the homodimer in the TMH2 and TMH3 regions [69], which also comes into agreement with our study.

### 3.3. M Protein Mutation Analysis

Regarding mutation analysis, from the 127,1550 genomes analyzed, 21,868 sequences carried SNPs at M protein dimer predicted interaction residues. This represents only 1.7% of all retrieved genomes suggesting that the predicted interfacial region is extremely conserved [71]. We identified 91 unique SNPs in this predicted interface. From these, 2.77% had a ΔΔG_binding_ higher than 0.50 kcal/mol, which means that these mutations can have a negative impact in the M protein dimer stability and 12.27% had a ΔΔG_binding_ lower than −0.50 kcal/mol, and, hence, could have a favorable impact in M protein dimer stability. Most mutations did not appear to influence M protein dimer interfacial stabilization, since about 85% showed ΔΔG_binding_ values between −0.50 kcal/mol and 0.50 kcal/mol. The ones that seem to lead to a gain of stabilization are presented in Table 9. We included here I82T as it is very close to our established threshold and is the most prevalent detected mutation. Most SNPs remained as non-polar residues (55.53%) or transitioned from non-polar to polar residues (41.68%) and most continued as non-aromatic residues. Since the M protein is a membrane protein, many non-polar residues were found within the membrane region, and, as such, most predicted interactions involved non-polar residues. However, mutations from non-polar to polar residues may confer a gain in conformation stability as they may establish hydrogen bonds. In our work, 9057 (99.36%) of non-polar to polar SNPs had ΔΔG_binding_ negative values, which endorses the maintenance or increase in stability as proposed. Mutations in homologous SARS-CoV experimentally interacting residues P59, W92, L93, and F96 were sparse and showed ΔΔG_binding_ values close to zero. Three exceptions were exposed: L93S and W92Q with ΔΔG_binding_ values lower than −0.5 kcal/mol, suggesting that these residues were also extremely important for M protein dimer interaction; and L93P ΔΔG_binding_ = 2.29 kcal/mol) value, the second highest, probably due to the destabilization caused by Proline in the TMH3 α-helix.

The most common mutations were I82T (28.88%) and V70L (28.82%), key residues for M monomers interaction as I82 and V70 interaction was conserved throughout the entire MDs simulation with a mean distance of 8.62 ± 0.65 Å for I82-V70 and 9.08 ± 0.66 Å for V70-I82 interactions (monomer A–monomer B). Both these residues (V70 and I82) had low RMSF values and were occluded from solvent upon complex formation (ΔSASA values between 45–88 Å2), which protects the established interactions. I82T and V70L, showed ΔΔG_binding_ values of −0.49 ± 0.38 kcal/mol and −0.02 ± 0.22 kcal/mol, suggesting that I82T is the most favorable, high-prevalent mutation and should be further studied. A previous work that established structure changes caused by M protein mutations, suggested that I82T and V70L mutations will not lead to a significant impact in M protein monomer secondary structure [72].

Overall, most represented clades in our mutation study were GRY (36.69%), containing VOC and GH (21.25%), G (19.06%), and GR (17.27%), containing VOC and VOI. This could mean that SNPs in the interface region may impact SARS-CoV-2 life cycle, specifically regarding the M protein functions. Furthermore, these mutations are intrinsically related to known VOC and VOIs. For instance, V70L and I82T mutations appeared in 99.5 and 97.64% of clades sequences that contain VOC and VOI. The most common mutation in VOC was V70L, detected in 6137 VOC genomes, and 97.35% of the time this mutation was detected, it appeared in pango lineage B.1.1.7, a VOC in clade GRY.

There were 25 co-occurring mutations on the GISAID data, 12 of which on interfacial residues involved in PPIs present throughout the entire MDs simulation. Even though SNP V70L only co-occurred with other mutations in nine cases, these sequences were from clade GRY, which contains several VOC. Overall, clades G (27.45%), GRY (23.53%), GH (23.53%), and GR (19.61%) were the most represented in our co-occurrence results, all containing VOC. V70L does not seem to be by itself relevant for homodimer formation but seems to be a catalyzer if co-occurring with other interfacial mutations as found in various VOCs. Clades GV (3.92%) and S (1.96%) also contained sequences with co-occurring mutations, and the remaining ones did not show any co-occurring mutations. It is possible to conclude that most co-occurring mutations were indeed in VOC and VOI containing clades.

One of the new strategies in drug development has been to develop peptides to interrupt transmembrane interactions in dimers [73]. As such, to predict druggability, regions of interest to the design of new drugs/peptides capable of inhibiting the formation of the M protein homodimer, we subjected 16 structures representative of the 16 clusters of the MD production phase to the well establish tool, FTMap [48,58,59,60]. The highest-ranking cluster from FTMap showed key interactions with predicted interfacial residues. Probes on this cluster interacted with residues F96, F103, S108, S111, and F112, all highly conserved residues. In fact, this cluster only established interaction with three residues with a higher number of mutations: F100 (98), M109 (1088), and I97 (228). I97 and F100 were the only ones for which 50 to 30% of the known mutations led to a stabilization of the homodimer. Our results point to some crucial interactions established by these residues: F96-F96, F100-F96, F100-F112, F103-F103, F103-S108, F103-S111, and F103-F112 (Table 1, Figure 4). As such, this region seems to be the best candidate area for the development of a new drug/peptide to inhibit SARS-CoV-2 M protein dimer formation. This zone was also a promising target in another approach that searched for druggable targets in the homodimeric structure [63].

A computational docking approach recently proposed the M protein heterodimer interactions with E and S protein [16]. In this study, residues W55, F96, and F103 were predicted as interacting residues in M-E PPI and Y71, and Y75 as interacting residues in M-S PPI [16]. In our work, these residues were also shown as interacting residues in the M protein homodimer, F96 as well as F103 are also present in the best drug target candidate region. This promiscuous region serves as a good candidate for experimental drug/peptide validation not only for the M protein homodimer, but also for M-E and M-S protein heterodimers, which further promotes its importance for the SARS-CoV-2 virus formation.

## 4. Materials and Methods

This work can be split into three main steps: M protein monomer membrane orientation prediction, M protein dimer 3D structure prediction, and mutation effect assessment in the homodimer interface. The overall workflow to accomplish these goals is illustrated in Figure 1.

### 4.1. M Protein Monomer Structure and Membrane Orientation

As there are no experimentally resolved structures for SARS-CoV-2 M protein dimer or monomer, and protein homology to other known 3D structures is reduced, we used AlphaFold’s [31] team proposed monomeric structure from YP_009724393.1 sequence. AlphaFold is a state-of-the-art Neural Network (NN)-based algorithm that predicts protein 3D structures from their sequence with a mean accuracy of 2.1 Å [74]. From all the 223 amino acids in the M protein, AlphaFold was able to confidently predict a structure encompassing residues 11 to 203, which were the ones studied and the results presented henceforth. M proteins can suffer glycosylation in order to regulate protein function [75,76], but this process has not yet been studied in detail [77]. To the best of our knowledge there is only one available in silico prediction of N5, N21, N41, N43, N117, N212, N203, and N216 as the N-glycosylation sites of the M protein [77]. However, other studies open the possibility that the M protein is not N- but instead O-glycosylated [78]. Due to the lack of confident experimental data and considering that the predicted residues are far away from the binding homodimeric interface for which we aimed to analyze potential gain/loss of stability, we decided to reduce the modeling uncertainty and neglect glycosylation at this stage. Six different web-based resources for membrane orientation prediction were used: OPM [32], TMpred [33], TMHM [34,35], PSIPRED [36,37], CCTOP [38,39], and SACSMEMSAT [40]. OPM database can predict protein structure within the lipid bilayer, and it optimizes position considering protein-membrane interactions [32]. TMpred predicts membrane-spanning regions and orientations from naturally occurring membrane proteins [33]. TMHMM correctly predicts membrane proteins’ α-helices positions with an accuracy of 77%, differentiating between soluble and membrane proteins [34,35]. PSIPRED predicts membrane protein secondary structure based on position-specific scoring matrices [36,37]. CCTOP predicts transmembrane topology using known experimental and computational membrane topologies [38,39]. SACSMEMSAT can predict protein secondary structure and membrane protein topology from well-defined membrane protein data [40].

We used MD simulations for the M monomer initial minimization considering each membrane orientation obtained via OPM, TMpred and TMHMM, PSIPRED, CCTOP, and SACSMEMSAT. MDs were performed using GROMACS [42,43] and the CHARMM36 force field [79]. Each system was built with CHARMM-GUI [41] membrane builder with TIP3 waters, 0.9 M Na^+^ and Cl^−^ ions and a bilayer membrane with POPC:POPE:PI:POPS:PSM:Cholesterol, in order to replicate human ER membrane [80], as M protein is translated and virus is assembled in this organelle. System size, water molecules, ion numbers, and lipid composition are described in Supplementary Table S3. Systems initial minimization was performed to remove bad contacts using the steepest descent algorithm. In this step, systems were heated with a Berendsen thermostat at 310 K in the canonical ensemble (NVT) over 7 ns, an adequate temperature to use in SARS-CoV-2 M protein MD simulations [81]. Pressure was kept constant at one bar with an isothermal–isobaric ensemble (NPT) for 20 ns with a semi-isotropic pressure coupling algorithm [82]. Long-range electrostatic interactions were treated by the fast smooth Particle-Mesh Ewald (PME) method [83]. RMSD analysis was conducted in Pymol, version 1.2r3pre with protein and transmembrane Cα residues to establish structural differences between initial MD structure (AlphaFold M protein prediction) and membrane orientation equilibrated results.

### 4.2. M Protein Dimer and Interface Prediction

OPM, TMpred, and TMHMM protein monomers were selected from system equilibration results and subjected to M protein dimer prediction. To guide the protein–protein docking we used known information on SARS-CoV M protein that has a 90.5% sequence identity and 90% homology with SARS-CoV-2 M protein [28]. Two equilibrated M protein monomers from each membrane orientation were used for dimer prediction using the docking tool HADDOCK [44], version 2.4, a protein quaternary structure predictor based on experimental data. Since M protein is a membrane protein and most homodimers are symmetric [84], water docking results were not considered and docking results with TMH2 and TMH3 non-crystallographic symmetry restraints were generated. To determine M protein monomer’s active residues, CPORT [85], a protein–protein residue interaction predictor at an atomic level, was used and only transmembrane residues predicted by this tool were considered for downstream steps. For each membrane predictor, 5000 dimer structures were generated in rigid body docking phase (it0) and 1000 structures for the semi-flexible refinement phase (it1). Monomer structures at the dimer docking decoys were superimposed with initial monomer membrane orientation prediction, and the 3D structures for which the angle between both superimposed monomer membranes was inferior to 1°, overlaid membranes, were selected for further analysis. Upon the selection of the most 20 promising HADDOCK dimers 3D structures, we extended our work towards interface interacting residues prediction. Protein Interfaces, Surfaces and Assemblies (PISA) [86], a web-based tool that resorts to chemical–physical principles for analyzing and modeling of macromolecular interactions, was used as a first predictor for dimer interface residues on all 20 dimer structures. Two dimers were chosen based on PISA results and their comparison with SARS-CoV’s M protein dimer experimental results, highlighted homologous SARS-CoV-2 residues W20, W58, P59, W92, Y95, F96, and C159 as important residues for dimer stabilization. Selected structures were further subjected to PRODIGY [87,88]. PRODIGY not only predicts dimer interacting residues, but also helps to determine if a protein interface is crystallographic or biological, the latter meaning that the predicted dimer is biologically relevant.

The final dimer system was built in a similar way as above-mentioned for M protein monomer MD simulations [80] (Supplementary Table S3). Three independent dimer system replicas of 0.5 μs MD simulations were produced with GROMACS (production phase). M protein dimer equilibration was performed as described in the previous section. MD simulations were performed with an isothermal–isobaric ensemble. Temperature coupling was done using a Nose–Hoover thermostat with a time constant of 1 ps. To maintain a constant pressure, a semi-isotropic Parrinello–Rahman barostat was used with a time constant of 5 ps and compressibility of 4.5 × 10^–5^ bar^−1^. Electrostatic interactions were performed with fast smooth Particle-Mesh Ewald, with a cutoff of 1.2 nm and Hydrogen bonds were constrained using the linear constraint solver.

Dimer system RMSD (between initial MD structure from HADDOCK TMHMM model and membrane orientation equilibrated results) and RMSF calculations were performed using Cα atoms with GROMACS package. CCA, which tracks the movements of two or more sets of time series data relative to one another, was performed using the Bio3D R package [89] based on the Cα atoms. SASA analysis for each residue was performed with the GROMACS package SASA analyses were performed for the dimer complex (SASA_complex_) and each monomer separately (SASA_monomerA_ and SASA_monomerB_), and ΔSASA was calculated for each residue as SASA_complex_ − (SASA_monomerA_ + SASA_monomerB_). ΔSASA values provide another quantitative measure of conformational change upon protein coupling. To further understand the behavior upon complex formation, we also calculated _rel_SASA for each residue that comes from the quotient between ΔSASA and SASA_monomer_. To detect possible interacting residues, a structure was retrieved every 2 ns, totaling 100 structures from 300 ns until 500 ns, for each replica as further explored in Supplementary Figures S2–S4. These structures were then submitted to an in-house script that detected residues for which side chains were within 5 Å of each other, using a 90% prevalence time as a cut-off.

### 4.3. M protein Mutation Analysis

Genome and protein sequences for this study were obtained from the GISAID [51] database (Accession Numbers are listed at Supplementary Information) and are available upon request at https://www.gisaid.org. MicroGMT [45], a python package, was developed, optimized, and used for SARS-CoV-2 M gene mutation analysis, to track indels and SNPs. This software requires raw or assembled genome sequences and works through database comparison to detect genomic mutations. Only non-synonymous SNPs at the M gene region for predicted interacting residues were considered for further studies. For M protein sequence mutation analysis, we used the Rahman et al. approach that works through pairwise analysis and comparison [46]. This method uses Multiple Sequence Alignment (MSA) and pairwise alignments to detect mutations in large datasets in a fast and accurate manner and has also been used in other studies regarding different SARS-CoV-2 proteins. Both tools were used with default parameters and all available sequences were compared against a reference, the first SARS-CoV-2 genome sequenced (NC_045512.2).

To determine the impact of mutations in M protein dimer stability, Gibbs energy difference was calculated using FoldX [47], an empirical force field. This approach evaluates the impact of mutations in protein stability through free energy variation )(ΔΔG_binding_ = ΔG_mutant_ − ΔG_WT_) between mutant protein and reference protein, considering contributions from hydrophobic, polar, Van der Waals, hydrogen bonds, and electrostatic interactions [47]. To avoid considering mean ΔΔG_binding_ values close to zero as relevant for protein stability, we established a low (below −0.5 kcal/mol) and high cut-off off (above 0.5 kcal/mol). Results for this step were analyzed considering residue polarities, both for the WT (protein sequence YP_009724393.1) and mutated proteins, as well as splitting residues by aromaticity, as both these characteristics have a major impact on protein–protein interactions. Residues considered as polar were R, N, D, C, E, N, H, K, S, T, Q, and Y; residues considered as non-polar were A, G, I, L, M, F, P, W, and V. Residues F, W, and Y were considered as aromatic.

To further identify ligand binding hotspots on SARS-CoV-2 M protein homodimer, an ensemble of representative structures was attained by clustering the production phase of the MD simulation. This clustering was performed by concatenating the trajectories and clustering with GROMACS using the gromos method with a cutoff of 0.25 nm. The 16 clusters were subjected to the FTMap [48,58,59,60] tool using the default parameters. FTMap [48,58,59,60] uses 16 small organic molecules as probes and samples/scores billions of positions to identify positions of interest to the development of new drugs.

All presented structure images were produced with Protein Imager [90], ggplot2 R package [91], and Bio3D R package [89].

## 5. Conclusions

As M protein dimer has several important functions during SARS-CoV-2 life cycle, it was fundamental to understand its structure–function relationship. Herein, upon establishing a comprehensive and well detailed computational pipeline, we were able not only to assess mutation effects at this interface but also to understand the specificities of the behavior of this region and establish the consequences for dimer stability. This was the first time that SARS-CoV-2 M protein dimer structure, interactions and mutational effects were proposed and thoroughly studied either computationally or experimentally. M protein is overall well conserved, showing that key-residues F96, F103, S108, S111, and F112 are preserved and able to form important interactions in the dimer. These residues can now be assessed as regions of interest for new therapeutic solutions regarding SARS-CoV-2.

## Figures and Tables

**Table 1:** SARS-CoV-2 M protein dimer interacting residues, using a prevalence time cut-off of 90% (all results were listed as mean values ± standard deviation).

Monomer A	ΔSASA A (Å^2^)	_rel_SASA A	Monomer B	ΔSASA B	_rel_SASA B	Percentage (%)	Cα Distance
				(Å^2^)			(Å)
W55	69.00 ± 23.91	0.62 ± 0.16	L93	70.51 ± 16.96	0.67 ± 0.14	100.00	10.93 ± 0.65
V66	57.26 ± 11.49	0.80 ± 0.13	V66	58.18 ± 11.81	0.80 ± 0.10	100.00	7.11 ± 0.32
A69	15.96 ± 6.92	0.93 ± 0.15	V70	87.47 ± 11.74	0.88 ± 0.08	100.00	6.31 ± 0.41
V70	83.79 ± 16.05	0.81 ± 0.16	A69	14.78 ± 9.16	0.78 ± 0.43	100.00	6.70 ± 0.50
V70	83.79 ± 16.05	0.81 ± 0.16	V70	87.47 ± 11.74	0.88 ± 0.08	100.00	5.25 ± 0.51
W75	66.06 ± 38.00	0.33 ± 0.18	Y71	8.53 ± 40.92	0.09 ± 0.57	100.00	11.42 ± 0.74
I82	63.02 ± 18.62	0.65 ± 0.14	V70	87.47 ± 11.74	0.88 ± 0.08	100.00	8.62 ± 0.65
W92	64.08 ± 12.99	0.87 ± 0.10	W92	48.02 ± 16.01	0.76 ± 0.17	100.00	12.58 ± 0.49
L93	67.87 ± 23.73	0.62 ± 0.20	P59	11.83 ± 23.49	0.20 ± 0.53	100.00	8.62 ± 0.61
F96	67.33 ± 16.53	0.90 ± 0.09	F96	52.22 ± 15.81	0.89 ± 0.12	100.00	9.67 ± 0.65
F103	66.38 ± 15.37	0.88 ± 0.10	F103	78.66 ± 15.91	0.95 ± 0.07	100.00	10.79 ± 0.58
M109	89.25 ± 27.84	0.54 ± 0.14	F103	78.66 ± 15.91	0.95 ± 0.07	100.00	8.31 ± 0.44
P59	32.47 ± 25.51	0.50 ± 0.27	L93	70.51 ± 16.96	0.67 ± 0.14	99.67	09.01 ± 0.62
F112	76.09 ± 25.84	0.84 ± 0.08	F100	64.39 ± 26.02	0.50 ± 0.19	99.67	9.13 ± 0.49
V70	83.79 ± 16.05	0.81 ± 0.16	I82	45.82 ± 20.01	0.50 ± 0.20	99.34	9.08 ± 0.66
F100	83.51 ± 28.35	0.62 ± 0.14	F112	38.18 ± 31.03	0.52 ± 0.41	99.34	9.16 ± 0.55
W55	69.00 ± 23.91	0.62 ± 0.16	I97	22.90 ± 22.99	0.23 ± 0.24	99.01	11.45 ± 0.68
W92	64.08 ± 12.99	0.87 ± 0.10	L93	70.51 ± 16.96	0.67 ± 0.14	99.01	11.78 ± 0.60
R107	71.92 ± 29.68	0.36 ± 0.13	M109	86.63 ± 28.09	0.49 ± 0.14	99.01	7.72 ± 0.77
L62	24.35 ± 18.78	0.44 ± 0.30	L62	17.37 ± 14.70	0.34 ± 0.30	98.35	11.78 ± 0.44
M109	89.25 ± 27.84	0.54 ± 0.14	F100	64.39 ± 26.02	0.50 ± 0.19	97.36	8.9 ± 0.57
M109	89.25 ± 27.84	0.54 ± 0.14	A104	17.84 ± 14.34	0.26 ± 0.21	97.36	7.78 ± 0.50
I82	63.02 ± 18.62	0.65 ± 0.14	L67	14.01 ± 19.51	0.17 ± 0.24	96.37	8.69 ± 0.55
F103	66.38 ± 15.37	0.88 ± 0.10	S108	9.79 ± 12.38	0.28 ± 0.76	95.05	10.8 ± 0.67
F112	76.09 ± 25.84	0.84 ± 0.08	F103	78.66 ± 15.91	0.95 ± 0.07	94.72	11.13 ± 0.55
W75	66.06 ± 38.00	0.33 ± 0.18	V70	87.47 ± 11.74	0.88 ± 0.08	94.39	10.58 ± 0.65
F103	66.38 ± 15.37	0.88 ± 0.10	F112	38.18 ± 31.03	0.52 ± 0.41	94.39	10.28 ± 0.69
I82	63.02 ± 18.62	0.65 ± 0.14	V66	58.18 ± 11.81	0.80 ± 0.10	93.07	9.02 ± 0.49
W55	69.00 ± 23.91	0.62 ± 0.16	F96	52.22 ± 15.81	0.89 ± 0.12	93.07	11.66 ± 0.63
V66	57.26 ± 11.49	0.80 ± 0.13	A85	0.92 ± 6.38	0.00 ± 0.00	92.08	9.78 ± 0.44
F103	66.38 ± 15.37	0.88 ± 0.10	S111	−3.07 ± 3.85	0.00 ± 0.00	92.08	11.02 ± 0.76
A85	1.49 ± 6.45	0.00 ± 0.00	V66	58.18 ± 11.81	0.80 ± 0.10	91.42	9.62 ± 0.45
F100	83.51 ± 28.35	0.62 ± 0.14	F96	52.22 ± 15.81	0.89 ± 0.12	91.42	11.66 ± 0.72
M109	89.25 ± 27.84	0.54 ± 0.14	R107	53.28 ± 38.80	0.26 ± 0.18	91.42	8.96 ± 0.68

**Table 2:** Mutation distribution in relation to ΔΔG_binding_.

ΔΔG_binding_ > 0.5 kcal/mol	ΔΔG_binding_ < −0.5 kcal/mol	−0.5 kcal/mol < ΔΔG_binding_
		<0.5 kcal/mol
606 (2.77%)	2683 (12.27%)	18,579 (84.96%)

**Table 3:** ΔΔG_binding_ average values for four different sets of residues according to polarity change.

	Polar Residue to Non-Polar Residue	Remained a Polar Residue	Non-Polar to a Polar Residue	Remained a Non-Polar Residue
Frequency	0.11%	2.68%	41.68%	55.53%
ΔΔG_binding_ (kcal/mol)	1.14 ± 0.48	0.65 ± 1.07	−0.42 ± 0.36	0.14 ± 0.49

**Table 4:** ΔΔG_binding_ average values for four different sets of residues according to aromaticity change.

	Remained an Aromatic Residue	Aromatic to a Non-Aromatic Residue	Non-Aromatic to an Aromatic Residue	Remained a Non-Aromatic Residue
Frequency	0.03%	2.69%	7.27%	90.01%
ΔΔG_binding_ (kcal/mol)	−0.20 ± 0.15	−0.11 ± 0.30	0.09 ± 0.29	0.04 ± 0.77

**Table 5:** ΔΔG_binding_ average values for the most frequent mutation found in each clade.

	GRY	GH	G	GR	GV	S	O	L	V
Frequency	36.69%	21.25%	19.06%	17.27%	4.36%	0.90%	0.38%	0.05%	0.05%
Most frequent mutation in clade (percentage)	V70L (73.30%)	I82T (47.23%)	I82T (71.26%)	V70F (26.32%)	-	-	-	-	-
ΔΔG_binding_ for most frequent mutation (kcal/mol)	−0.02 ± 0.22	−0.49 ± 0.38	−0.49 ± 0.38	0.17 ± 0.47	-	-	-	-	-

**Table 6:** ΔSASA and relSASA for highlighted residues.

Residue	ΔSASA (Å^2^)	relSASA
I82	54.42 ± 13.27	0.58 ± 0.12
V70	85.63 ± 11.01	0.84 ± 0.10
A69	15.37 ± 4.26	0.90 ± 0.12
M109	87.94 ± 14.46	0.52 ± 0.07
A104	13.69 ± 8.89	0.21 ± 0.13
R107	62.60 ± 21.03	0.32 ± 0.10
W75	49.28 ± 20.33	0.27 ± 0.11

**Table 7:** Summary for predicted interfacial residues that interact with the first probe.

Residue	Number of Mutations	ΔΔG_binding_ < −0.5 kcal/mol	−0.5 kcal/mol < ΔΔG_binding_ < 0.5 kcal/mol	ΔΔG_binding_ > 0.5 kcal/mol
F96	1	0%	100%	0%
S111	1	0%	0%	100%
S108	2	0%	0%	100%
F103	7	0%	100%	0%
F112	14	0%	100%	0%
W55	32	0%	100%	0%
F100	98	30%	70%	0%
I97	228	50%	50%	0%
M109	1088	0%	100%	0%

**Table 8:** Summary for predicted interfacial residues that interact with the second probe.

Residue	Number of Mutations	ΔΔG_binding_ < −0.5 kcal/mol	−0.5 kcal/mol < ΔΔG_binding_ < 0.5 kcal/mol	ΔΔG_binding_ > 0.5 kcal/mol
P59	4	25%	75%	0%
W92	4	25%	75%	0%
L62	6	50%	50%	0%
L67	40	17%	66%	17%
L93	61	1%	98%	1%
V66	502	0%	100%	0%
A85	1604	75%	25%	0%
I82	7094	10%	90%	0%

**Table 9:** M protein mutations that resulted in a stabilization gain of the homodimer structure.

Mutation	ΔΔG_binding_ (kcal/mol)
I82T	−0.49
I97T	−0.5
I82S	−0.55
W92Q	−0.59
L62S	−0.62
A104T	−0.63
I97S	−0.74
L93S	−0.76
F100S	−0.78
P59Q	−0.83
Y71H	−0.9
A104S	−0.91
A69T	−0.92
A85S	−0.93
L67H	−1.07
A69S	−1.2

## Data Availability

The genomic datasets analyzed during the current study are freely available in the GISAID repository, at https://www.gisaid.org/ (accessed on 3 May 2021), and Accessions Numbers are available at Supplementary Information. GISAID has an application procedure for obtaining access to the data, which should be followed for any researcher that wants to use it. Detailed data analysis results are also available at Supplementary Materials. Any material requests should be addressed to I.S.M.: irina.moreira@cnc.uc.pt.

## Supplementary Materials

The following supporting information can be downloaded at: https://www.mdpi.com/article/10.3390/ijms23062986/s1.
