# Supplementary material for: SARS-CoV-2 Membrane Protein: From Genomic Data to Structural New Insights
Source: Int J Mol Sci. 2022 Mar 10;23(6):2986. doi: 10.3390/ijms23062986 (PMC8948900; doi:10.3390/ijms23062986)
Supplement: Supplementary file 1 [file ijms-23-02986-s001.zip › Supplementary Tables and Figures.pdf]

Supplementary Material

SARS-CoV-2 Membrane Protein: From Genomic Data to Structural New Insights

Table S1. Analysis for each detected mutation in the predicted interface residues for SARS-CoV-2 M protein. This table describes the frequency of each mutation (Frequency), their mean $\Delta\Delta G_{binding}$ values ($\Delta\Delta G$), RMSF for each original residue (RMSF), solvent-accessible surface area (SASA) for each original residue in the complex ($SASA_{cpx}$), SASA for each original residue in the monomer ($SASA_{mon}$), ΔSASA for each original residue (ΔSASA), relative SASA for each original residue (${}_{rel}SASA$), the number of interactions each original residues establish (Interactions) and the distribution of the mutation across the GISAID clades (Clade) (all the presented results are mean values ± standard deviation).

Mutation	Frequency	ΔΔG	RMSF	$SASA_{cpx}$	$SASA_{mon}$	ΔSASA	${}_{rel}$SASA	Interactions	Clade (%)								
		Mean ± SD	Mean ± SD	Mean ± SD	Mean ± SD	Mean ± SD	Mean ± SD		L	S	V	G	GH	GR	GV	GRY	O
I82T	6316	−0.49 ± 0.38	91.22 ± 19.94	38.67 ± 15.10	93.09 ± 13.33	54.42 ± 13.27	0.58 ± 0.12	4	0.06	0.24	0.00	47.02	34.74	14.06	1.71	1.82	0.35
V70L	6303	−0.021 ± 0.22	86.27 ± 18.53	15.82 ± 13.93	101.45 ± 11.56	85.63 ± 11.01	0.84 ± 0.10	6	0.00	0.00	0.00	0.68	1.08	4.43	0.21	93.30	0.30
V70F	1455	0.17 ± 0.47	86.27 ± 18.53	15.82 ± 13.93	101.45 ± 11.56	85.63 ± 11.01	0.84 ± 0.10	6	0.00	0.27	0.20	4.95	7.90	68.32	6.25	11.62	0.49
A85S	1215	−0.93 ± 0.30	79.81 ± 13.46	6.65 ± 4.25	7.85 ± 4.73	1.21 ± 1.56	0.14 ± 0.17	2	0.00	0.25	0.00	4.44	85.03	3.62	1.15	6.26	0.24
M109I	1005	0.05 ± 0.20	118.10 ± 20.29	80.28 ± 22.41	168.22 ± 20.18	87.94 ± 14.46	0.52 ± 0.07	5	0.00	0.10	0.00	4.38	54.03	13.03	8.56	19.50	0.40
A104V	959	−0.09 ± 0.19	105.19 ± 27.34	48.72 ± 14.21	62.41 ± 13.29	13.69 ± 8.89	0.21 ± 0.13	1	0.00	0.00	0.00	9.28	10.22	16.16	39.31	24.82	0.21
I82S	712	−0.55 ± 0.37	91.22 ± 19.94	38.67 ± 15.10	93.09 ± 13.33	54.42 ± 13.27	0.58 ± 0.12	4	0.00	14.75	0.00	80.20	2.25	0.98	0.14	1.68	0.00
A69S	574	−1.12 ± 0.23	83.55 ± 16.47	1.59 ± 3.05	16.96 ± 6.77	15.37 ± 4.26	0.90 ± 0.12	2	0.17	0.17	0.00	9.93	20.38	23.34	11.85	33.28	0.88
R107H	566	1.15 ± 0.68	122.51 ± 37.92	134.74 ± 27.60	197.33 ± 18.99	62.60 ± 21.03	0.32 ± 0.10	2	0.35	0.18	0.00	1.77	13.78	45.23	2.65	36.04	0.00
V70I	526	−0.06 ± 0.09	86.27 ± 18.53	15.82 ± 13.93	101.45 ± 11.56	85.63 ± 11.01	0.84 ± 0.10	6	0.19	3.80	0.19	1.33	3.42	76.81	8.75	5.51	0.00
V66L	430	−0.06 ± 0.05	72.96 ± 15.00	14.52 ± 8.56	72.23 ± 9.42	57.72 ± 5.87	0.80 ± 0.07	4	0.47	0.00	0.00	8.37	9.77	36.74	10.00	33.95	0.70
W75L	382	0.04 ± 0.30	134.37 ± 26.17	136.35 ± 44.73	185.63 ± 24.99	49.28 ± 20.33	0.27 ± 0.11	2	0.00	10.21	1.05	6.28	10.99	14.40	3.93	53.14	0.00
A85V	352	−0.17 ± 0.07	79.81 ± 13.46	6.65 ± 4.25	7.85 ± 4.73	1.21 ± 1.56	0.14 ± 0.17	2	0.00	0.28	0.00	10.23	16.48	14.20	2.27	56.53	0.00
A69V	158	−0.15 ± 0.08	83.55 ± 16.47	1.59 ± 3.05	16.96 ± 6.77	15.37 ± 4.26	0.90 ± 0.12	2	0.63	1.90	1.90	22.78	9.49	24.68	5.06	32.91	0.63
I97T	116	−0.50 ± 0.46	89.75 ± 19.33	77.20 ± 19.03	96.61 ± 17.39	19.41 ± 6.87	0.20 ± 0.06	1	0.00	0.00	0.00	0.86	12.93	10.34	6.03	69.83	0.00
F100L	64	0.02 ± 0.10	92.63 ± 24.89	55.87 ± 22.80	129.82 ± 17.14	73.95 ± 16.79	0.57 ± 0.10	4	0.00	0.00	0.00	25.00	6.25	9.38	9.38	50.00	0.00
I82V	61	0.05 ± 0.05	91.22 ± 19.94	38.67 ± 15.10	93.09 ± 13.33	54.42 ± 13.27	0.58 ± 0.12	4	0.00	0.00	0.00	47.54	1.64	42.62	6.56	1.64	0.00
I97F	56	−0.01 ± 0.28	89.75 ± 19.33	77.20 ± 19.03	96.61 ± 17.39	19.41 ± 6.87	0.20 ± 0.06	1	0.00	0.00	0.00	23.21	5.36	8.93	12.50	50.00	0.00
L93F	48	−0.02 ± 0.10	80.02 ± 12.90	38.00 ± 19.35	107.19 ± 12.26	69.19 ± 9.59	0.65 ± 0.08	4	0.00	0.00	0.00	18.75	37.50	20.83	0.00	20.83	2.08

I97V	47	0.08 ± 0.11	89.75 ± 19.33	77.20 ± 19.03	96.61 ± 17.39	19.41 ± 6.87	0.20 ± 0.06	1	0.00	0.00	0.00	4.26	14.89	68.09	0.00	8.51	4.26
V70A	45	0.16 ± 0.12	86.27 ± 18.53	15.82 ± 13.93	101.45 ± 11.56	85.63 ± 11.01	0.84 ± 0.10	6	0.00	2.22	0.00	26.67	51.11	6.67	0.00	0.00	13.33
V66M	40	−0.07 ± 0.06	72.96 ± 15.00	14.52 ± 8.56	72.23 ± 9.42	57.72 ± 5.87	0.80 ± 0.07	4	0.00	0.00	0.00	5.00	40.00	17.50	0.00	37.50	0.00
M109L	38	0.04 ± 0.16	118.10 ± 20.29	80.28 ± 22.41	168.22 ± 20.18	87.94 ± 14.46	0.52 ± 0.07	5	0.00	0.00	0.00	0.00	7.89	13.16	2.63	76.32	0.00
M109V	38	0.08 ± 0.28	118.10 ± 20.29	80.28 ± 22.41	168.22 ± 20.18	87.94 ± 14.46	0.52 ± 0.07	5	0.00	0.00	0.00	13.16	31.58	21.05	2.63	31.58	0.00
A85T	30	−0.49 ± 0.30	79.81 ± 13.46	6.65 ± 4.25	7.85 ± 4.73	1.21 ± 1.56	0.14 ± 0.17	2	0.00	0.00	0.00	3.33	23.33	3.33	10.00	60.00	0.00
F100S	29	−0.78 ± 0.33	92.63 ± 24.89	55.87 ± 22.80	129.82 ± 17.14	73.95 ± 16.79	0.57 ± 0.10	4	0.00	0.00	0.00	6.90	68.97	13.79	0.00	10.34	0.00
W75S	28	0.02 ± 0.32	134.37 ± 26.17	136.35 ± 44.73	185.63 ± 24.99	49.28 ± 20.33	0.27 ± 0.11	2	0.00	0.00	0.00	3.57	14.29	21.43	0.00	60.71	0.00
R107L	21	1.85 ± 0.51	122.51 ± 37.92	134.74 ± 27.60	197.33 ± 18.99	62.60 ± 21.03	0.32 ± 0.10	2	0.00	0.00	0.00	9.52	42.86	23.81	4.76	14.29	4.76
L67F	21	0.02 ± 0.08	76.67 ± 17.80	65.01 ± 14.59	80.66 ± 13.06	15.65 ± 5.30	0.19 ± 0.06	1	0.00	0.00	0.00	9.52	33.33	14.29	19.05	23.81	0.00
V66A	21	0.21 ± 0.08	72.96 ± 15.00	14.52 ± 8.56	72.23 ± 9.42	57.72 ± 5.87	0.80 ± 0.07	4	0.00	0.00	0.00	4.76	66.67	9.52	4.76	14.29	0.00
W55L	20	−0.03 ± 0.13	90.56 ± 16.30	45.24 ± 23.00	112.80 ± 19.40	67.56 ± 16.61	0.60 ± 0.15	3	0.00	0.00	0.00	0.00	10.00	35.00	0.00	55.00	0.00
L67I	12	−0.04 ± 0.12	76.67 ± 171.80	65.01 ± 14.59	80.66 ± 13.06	15.65 ± 5.30	0.19 ± 0.06	1	0.00	0.00	0.00	8.33	91.67	0.00	0.00	0.00	0.00
W55C	12	−0.13 ± 0.20	90.56 ± 16.30	45.24 ± 23.00	112.80 ± 19.40	67.56 ± 16.61	0.60 ± 0.15	3	0.00	0.00	0.00	25.00	8.33	8.33	0.00	58.33	0.00
A104S	10	−0.91 ± 0.38	105.19 ± 27.34	48.72 ± 14.21	62.41 ± 13.29	13.69 ± 8.89	0.21 ± 0.13	1	0.00	0.00	0.00	0.00	60.00	10.00	10.00	20.00	0.00
Y71H	10	−0.90 ± 0.50	92.35 ± 19.94	56.84 ± 28.93	84.97 ± 34.74	28.12 ± 10.60	0.34 ± 0.13	1	0.00	0.00	0.00	0.00	30.00	0.00	10.00	60.00	0.00
W75R	9	0.05 ± 0.33	134.37 ± 26.17	136.35 ± 44.73	185.63 ± 24.99	49.28 ± 20.33	0.27 ± 0.11	2	0.00	0.00	0.00	33.33	33.33	11.11	0.00	22.22	0.00
V66G	9	0.32 ± 0.10	72.96 ± 15.00	14.52 ± 8.56	72.23 ± 9.42	57.72 ± 5.87	0.80 ± 0.07	4	0.00	0.00	0.00	11.11	33.33	44.44	0.00	0.00	11.11
L93M	8	−0.01 ± 0.07	80.02 ± 12.90	38.00 ± 19.35	107.19 ± 12.26	69.19 ± 9.59	0.65 ± 0.08	4	0.00	0.00	0.00	0.00	0.00	0.00	100.00	0.00	0.00
F112L	7	0.01 ± 0.28	103.70 ± 34.74	23.38 ± 21.40	80.51 ± 27.54	57.14 ± 10.26	0.73 ± 0.15	4	0.00	0.00	0.00	28.57	28.57	0.00	0.00	28.57	14.29
M109T	7	0.08 ± 0.28	118.10 ± 20.29	80.28 ± 22.41	168.22 ± 20.18	87.94 ± 14.46	0.52 ± 0.07	5	0.00	0.00	0.00	14.29	28.57	0.00	0.00	57.14	0.00
R107C	6	1.78 ± 0.49	122.51 ± 37.92	134.74 ± 27.60	197.33 ± 18.99	62.60 ± 21.03	0.32 ± 0.10	2	0.00	0.00	0.00	0.00	16.67	50.00	16.67	16.67	0.00
I97M	6	−0.12 ± 0.31	89.75 ± 19.33	77.20 ± 19.03	96.61 ± 17.39	19.41 ± 6.87	0.20 ± 0.06	1	0.00	0.00	0.00	0.00	0.00	66.67	0.00	33.33	0.00
A85D	6	−0.06 ± 0.07	79.81 ± 13.46	6.65 ± 4.25	7.85 ± 4.73	1.21 ± 1.56	0.14 ± 0.17	2	0.00	0.00	0.00	16.67	83.33	0.00	0.00	0.00	0.00
F112Y	5	−0.09 ± 0.27	103.70 ± 34.74	23.38 ± 21.40	80.51 ± 27.54	57.14 ± 10.26	0.73 ± 0.15	4	0.00	0.00	0.00	0.00	40.00	20.00	0.00	40.00	0.00
W75C	5	0.03 ± 0.28	134.37 ± 26.17	136.35 ± 44.73	185.63 ± 24.99	49.28 ± 20.33	0.27 ± 0.11	2	0.00	0.00	0.00	20.00	80.00	0.00	0.00	0.00	0.00
A69T	5	−0.92 ± 0.34	83.55 ± 16.47	1.59 ± 3.05	16.96 ± 6.77	15.37 ± 4.26	0.90 ± 0.12	2	0.00	0.00	0.00	0.00	0.00	0.00	20.00	80.00	0.00
A69P	5	1.00 ± 0.71	83.55 ± 16.47	1.59 ± 3.05	16.96 ± 6.77	15.37 ± 4.26	0.90 ± 0.12	2	0.00	0.00	0.00	0.00	0.00	0.00	100.00	0.00	0.00
L67Y	5	0.01 ± 0.11	76.67 ± 17.80	65.01 ± 14.59	80.66 ± 13.06	15.65 ± 5.30	0.19 ± 0.06	1	0.00	0.00	0.00	20.00	20.00	0.00	20.00	20.00	20.00
A104T	4	−0.63 ± 0.44	105.19 ± 27.34	48.72 ± 14.21	62.41 ± 13.29	13.69 ± 8.89	0.21 ± 0.13	1	0.00	0.00	0.00	0.00	0.00	50.00	25.00	25.00	0.00

F103Y	4	−0.31 ± 0.36	95.69 ± 21.66	6.41 ± 6.87	78.93 ± 14.74	72.52 ± 8.95	0.92 ± 0.04	6	0.00	0.00	0.00	0.00	50.00	25.00	0.00	25.00	0.00
F100C	3	0.07 ± 0.21	92.63 ± 24.89	55.87 ± 22.80	129.82 ± 17.14	73.95 ± 16.79	0.57 ± 0.10	4	0.00	0.00	0.00	0.00	0.00	0.00	100.00	0.00	0.00
I82M	3	0.00 ± 0.06	91.22 ± 19.94	38.67 ± 15.10	93.09 ± 13.33	54.42 ± 13.27	0.58 ± 0.12	4	0.00	0.00	0.00	0.00	0.00	100.00	0.00	0.00	0.00
Y71C	3	0.05 ± 0.18	92.35 ± 19.94	56.84 ± 28.93	84.97 ± 34.74	28.12 ± 10.60	0.34 ± 0.13	1	0.00	66.67	0.00	0.00	33.33	0.00	0.00	0.00	0.00
L62S	3	−0.62 ± 0.34	71.67 ± 9.75	25.27 ± 7.76	46.13 ± 12.86	20.86 ± 7.36	0.45 ± 0.12	1	0.00	0.00	0.00	0.00	33.33	66.67	0.00	0.00	0.00
L62I	3	0.03 ± 0.09	71.67 ± 9.75	25.27 ± 7.76	46.13 ± 12.86	20.86 ± 7.36	0.45 ± 0.12	1	0.00	0.00	0.00	0.00	33.33	0.00	33.33	33.33	0.00
R107S	2	1.21 ± 0.62	122.51 ± 37.92	134.74 ± 27.60	197.33 ± 18.99	62.60 ± 21.03	0.32 ± 0.10	2	0.00	0.00	0.00	0.00	100.00	0.00	0.00	0.00	0.00
F103V	2	0.05 ± 0.40	95.69 ± 21.66	6.41 ± 6.87	78.93 ± 14.74	72.52 ± 8.95	0.92 ± 0.04	6	0.00	0.00	0.00	50.00	0.00	50.00	0.00	0.00	0.00
I97R	2	−0.31 ± 0.55	89.75 ± 19.33	77.20 ± 19.03	96.61 ± 17.39	19.41 ± 6.87	0.20 ± 0.06	1	0.00	0.00	0.00	0.00	0.00	100.00	0.00	0.00	0.00
L93P	2	2.29 ± 0.30	80.02 ± 12.90	38.00 ± 19.35	107.19 ± 12.26	69.19 ± 9.59	0.65 ± 0.08	4	0.00	0.00	0.00	0.00	0.00	0.00	0.00	50.00	50.00
A69D	2	−0.14 ± 0.20	83.55 ± 16.47	1.59 ± 3.05	16.96 ± 6.77	15.37 ± 4.26	0.90 ± 0.12	2	0.00	0.00	0.00	50.00	0.00	0.00	0.00	0.00	50.00
V66F	2	−0.10 ± 0.07	72.96 ± 15.00	14.52 ± 8.56	72.23 ± 9.42	57.72 ± 5.87	0.80 ± 0.07	4	0.00	0.00	0.00	0.00	0.00	0.00	50.00	50.00	0.00
F112C	1	0.02 ± 0.32	103.70 ± 34.74	23.38 ± 21.40	80.51 ± 27.54	57.14 ± 10.26	0.73 ± 0.15	4	0.00	0.00	0.00	0.00	0.00	100.00	0.00	0.00	0.00
F112S	1	−0.34 ± 0.45	103.70 ± 34.74	23.38 ± 21.40	80.51 ± 27.54	57.14 ± 10.26	0.73 ± 0.15	4	0.00	0.00	0.00	0.00	0.00	100.00	0.00	0.00	0.00
S111L	1	0.99 ± 0.47	102.15 ± 24.07	1.94 ± 3.03	3.21 ± 4.80	1.27 ± 1.80	0.34 ± 0.36	1	0.00	0.00	0.00	100.00	0.00	0.00	0.00	0.00	0.00
S108F	1	0.82 ± 0.55	120.22 ± 27.48	13.54 ± 7.55	18.53 ± 8.82	4.98 ± 4.53	0.27 ± 0.23	1	0.00	0.00	0.00	0.00	0.00	100.00	0.00	0.00	0.00
S108A	1	0.89 ± 0.30	120.22 ± 27.48	13.54 ± 7.55	18.53 ± 8.82	4.98 ± 4.53	0.27 ± 0.23	1	0.00	0.00	0.00	0.00	0.00	0.00	100.00	0.00	0.00
A104L	1	−0.15 ± 0.17	105.19 ± 27.34	48.72 ± 14.21	62.41 ± 13.29	13.69 ± 8.89	0.21 ± 0.13	1	0.00	0.00	0.00	100.00	0.00	0.00	0.00	0.00	0.00
F103L	1	0.02 ± 0.35	95.69 ± 21.66	6.41 ± 6.87	78.93 ± 14.74	72.52 ± 8.95	0.92 ± 0.04	6	0.00	0.00	0.00	0.00	100.00	0.00	0.00	0.00	0.00
F100V	1	0.05 ± 0.18	92.63 ± 24.89	55.87 ± 22.80	129.82 ± 17.14	73.95 ± 16.79	0.57 ± 0.10	4	0.00	0.00	0.00	0.00	0.00	100.00	0.00	0.00	0.00
F100I	1	−0.03 ± 0.15	92.63 ± 24.89	55.87 ± 22.80	129.82 ± 17.14	73.95 ± 16.79	0.57 ± 0.10	4	0.00	0.00	0.00	0.00	0.00	100.00	0.00	0.00	0.00
I97S	1	−0.74 ± 0.42	89.75 ± 19.33	77.20 ± 19.03	96.61 ± 17.39	19.41 ± 6.87	0.20 ± 0.06	1	0.00	0.00	0.00	0.00	0.00	100.00	0.00	0.00	0.00
F96L	1	−0.05 ± 0.11	85.95 ± 21.44	13.54 ± 7.55	18.53 ± 8.82	4.98 ± 4.53	0.27 ± 0.23	3	0.00	0.00	0.00	0.00	0.00	0.00	0.00	0.00	100.00
L93S	1	−0.76 ± 0.30	80.02 ± 12.90	38.00 ± 19.35	107.19 ± 12.26	69.19 ± 9.59	0.65 ± 0.08	4	0.00	0.00	0.00	0.00	0.00	0.00	100.00	0.00	0.00
L93R	1	−0.47 ± 0.62	80.02 ± 12.90	38.00 ± 19.35	107.19 ± 12.26	69.19 ± 9.59	0.65 ± 0.08	4	0.00	0.00	0.00	0.00	0.00	100.00	0.00	0.00	0.00
L93I	1	−0.06 ± 0.08	80.02 ± 12.90	38.00 ± 19.35	107.19 ± 12.26	69.19 ± 9.59	0.65 ± 0.08	4	0.00	0.00	0.00	100.00	0.00	0.00	0.00	0.00	0.00
W92G	1	0.34 ± 0.23	77.61 ± 11.10	11.91 ± 8.36	67.96 ± 16.30	56.05 ± 9.67	0.83 ± 0.08	2	0.00	0.00	0.00	0.00	0.00	100.00	0.00	0.00	0.00
W92L	1	−0.03 ± 0.12	77.61 ± 11.10	11.91 ± 8.36	67.96 ± 16.30	56.05 ± 9.67	0.83 ± 0.08	2	0.00	0.00	0.00	0.00	0.00	0.00	0.00	100.00	0.00
W92R	1	−0.32 ± 0.49	77.61 ± 11.10	11.91 ± 8.36	67.96 ± 16.30	56.05 ± 9.67	0.83 ± 0.08	2	0.00	0.00	0.00	0.00	0.00	0.00	0.00	0.00	100.00
W92Q	1	−0.59 ± 0.49	77.61 ± 11.10	11.91 ± 8.36	67.96 ± 16.30	56.05 ± 9.67	0.83 ± 0.08	2	0.00	0.00	0.00	100.00	0.00	0.00	0.00	0.00	0.00

A85G	1	0.13 ± 0.06	79.81 ± 13.46	6.65 ± 4.25	7.85 ± 4.73	1.21 ± 1.56	0.14 ± 0.17	2	0.00	0.00	0.00	0.00	0.00	100.00	0.00	0.00	0.00
I82F	1	0.06 ± 0.10	91.22 ± 19.94	38.67 ± 15.10	93.09 ± 13.33	54.42 ± 13.27	0.58 ± 0.12	4	0.00	0.00	0.00	0.00	0.00	100.00	0.00	0.00	0.00
I82A	1	0.15 ± 0.07	91.22 ± 19.94	38.67 ± 15.10	93.09 ± 13.33	54.42 ± 13.27	0.58 ± 0.12	4	0.00	0.00	0.00	0.00	0.00	100.00	0.00	0.00	0.00
W75G	1	0.09 ± 0.32	134.37 ± 26.17	11.91 ± 8.36	67.96 ± 16.30	56.05 ± 9.67	0.83 ± 0.08	2	0.00	0.00	0.00	0.00	0.00	100.00	0.00	0.00	0.00
A69F	1	−0.16 ± 0.31	83.55 ± 16.47	1.59 ± 3.05	16.96 ± 6.77	15.37 ± 4.26	0.90 ± 0.12	2	0.00	0.00	0.00	0.00	0.00	0.00	0.00	100.00	0.00
A69G	1	0.16 ± 0.07	83.55 ± 16.47	1.59 ± 3.05	16.96 ± 6.77	15.37 ± 4.26	0.90 ± 0.12	2	0.00	0.00	0.00	100.00	0.00	0.00	0.00	0.00	0.00
L67H	1	−1.07 ± 0.43	76.67 ± 17.80	65.01 ± 14.59	80.66 ± 13.06	15.65 ± 5.30	0.19 ± 0.06	1	0.00	0.00	0.00	0.00	100.00	0.00	0.00	0.00	0.00
L67P	1	2.37 ± 0.34	76.67 ± 17.80	65.01 ± 14.59	80.66 ± 13.06	15.65 ± 5.30	0.19 ± 0.06	1	0.00	0.00	0.00	0.00	0.00	100.00	0.00	0.00	0.00
P59S	1	−0.30 ± 0.30	80.69 ± 16.51	27.90 ± 14.82	50.05 ± 18.94	22.15 ± 6.64	0.45 ± 0.13	2	0.00	0.00	0.00	0.00	0.00	100.00	0.00	0.00	0.00
P59L	1	−0.02 ± 0.08	80.69 ± 16.51	27.90 ± 14.82	50.05 ± 18.94	22.15 ± 6.64	0.45 ± 0.13	2	0.00	0.00	0.00	0.00	0.00	100.00	0.00	0.00	0.00
P59Q	1	−0.83 ± 0.35	80.69 ± 16.51	27.90 ± 14.82	50.05 ± 18.94	22.15 ± 6.64	0.45 ± 0.13	2	0.00	0.00	0.00	0.00	100.00	0.00	0.00	0.00	0.00
P59T	1	−0.14 ± 0.24	80.69 ± 16.51	27.90 ± 14.82	50.05 ± 18.94	22.15 ± 6.64	0.45 ± 0.13	2	0.00	0.00	0.00	0.00	100.00	0.00	0.00	0.00	0.00

Table S2. Analysis for detected co-occurring mutations in the predicted interface residues for SARS-CoV-2 M protein. This table describes the frequency of co-occurring mutations (Frequency), their $\Delta\Delta G_{binding}$ values ($\Delta\Delta G$), RMSF for each original residue (RMSF), solvent-accessible surface area (SASA) for each original residue in the complex ($SASA_{cpx}$), SASA for each original residue in the monomer ($SASA_{mon}$), ΔSASA for each original residue (ΔSASA), relative SASA for each original residue ($_{rel}SASA$), the number of interactions each original residues establish (Interactions) and the distribution of co-occurring mutations across the GISAID clades (Clade) (all the presented results are mean values ± standard deviation).

Co-occurrence	Frequency	Mutation	ΔΔG	RMSF	$SASA_{cpx}$	$SASA_{mon}$	ΔSASA	$_{rel}$ SASA	Interactions	Clade (%)								
			Mean ± SD	Mean ± SD	Mean ± SD	Mean ± SD	Mean ± SD	Mean ± SD		L	S	V	G	GH	GR	GV	GRY	O
M109L, V70L	8	M109L	0.03 ± 0.35	118 ± 20	80.28 ± 22.41	168.22 ± 20.18	87.94 ± 14.46	0.52 ± 0.07	5	0.00	0.00	0.00	0.00	0.00	0.00	0.00	100.00	0.00
		V70L		86 ± 19	15.82 ± 13.93	101.45 ± 11.56	85.63 ± 11.01	0.84 ± 0.10	6									
I82T, L93F	7	I82T	−0.5 ± 0.41	91 ± 20	38.67 ± 15.10	93.09 ± 13.33	54.42 ± 13.27	0.58 ± 0.12	4	0.00	0.00	0.00	0.00	100.00	0.00	0.00	0.00	0.00
		L93F		80 ± 13	38.00 ± 19.35	107.19 ± 12.26	69.19 ± 9.59	0.65 ± 0.08	4									
I82T, R107L	4	I82T	1.35 ± 0.64	91 ± 20	38.67 ± 15.10	93.09 ± 13.33	54.42 ± 13.27	0.58 ± 0.12	4	0.00	0.00	0.00	100.00	0.00	0.00	0.00	0.00	0.00
		R107L		123 ± 38	134.74 ± 27.60	197.33 ± 18.99	62.60 ± 21.03	0.32 ± 0.10	2									
A85S, V70F	3	A85S	−0.72 ± 0.64	80 ± 13	6.65 ± 4.25	7.85 ± 4.73	1.21 ± 1.56	0.14 ± 0.17	2	0.00	0.00	0.00	0.00	0.00	100.00	0.00	0.00	0.00
		V70F		86 ± 19	15.82 ± 13.93	101.45 ± 11.56	85.63 ± 11.01	0.84 ± 0.10	6									
A104V, V70L	3	A104V	−0.10 ± 0.32	105 ± 27	48.72 ± 14.21	62.41 ± 13.29	13.69 ± 8.89	0.21 ± 0.13	1	0.00	0.00	0.00	0.00	33.33	0.00	0.00	66.67	0.00

		V70L		86 ± 19	15.82 ± 13.93	101.45 ± 11.56	85.63 ± 11.01	0.84 ± 0.10	6									
I82T, M109V	2	I82T	−0.42 ± 0.50	91 ± 20	38.67 ± 15.10	93.09 ± 13.33	54.42 ± 13.27	0.58 ± 0.12	4	0.00	0.00	0.00	0.00	100.00	0.00	0.00	0.00	0.00
		M109V		118 ± 20	80.28 ± 22.41	168.22 ± 20.18	87.94 ± 14.46	0.52 ± 0.07	5									
I82T, V70F	2	I82T	−0.22 ± 0.63	91 ± 20	38.67 ± 15.10	93.09 ± 13.33	54.42 ± 13.27	0.58 ± 0.12	4	0.00	0.00	0.00	100.00	0.00	0.00	0.00	0.00	0.00
		V70F		86 ± 19	15.82 ± 13.93	101.45 ± 11.56	85.63 ± 11.01	0.84 ± 0.10	6									
I82T, M109I	2	I82T	−0.43 ± 0.44	91 ± 20	38.67 ± 15.10	93.09 ± 13.33	54.42 ± 13.27	0.58 ± 0.12	4	0.00	0.00	0.00	100.00	0.00	0.00	0.00	0.00	0.00
		M109I		118 ± 20	80.28 ± 22.41	168.22 ± 20.18	87.94 ± 14.46	0.52 ± 0.07	5									
I82T, V66M	2	I82T	−0.5 ± 0.37	91 ± 20	38.67 ± 15.10	93.09 ± 13.33	54.42 ± 13.27	0.58 ± 0.12	4	0.00	0.00	0.00	50.00	0.00	50.00	0.00	0.00	0.00
		V66M		73 ± 15	14.52 ± 8.56	72.23 ± 9.42	57.72 ± 5.87	0.80 ± 0.07	4									
A69V, A85S	2	A69V	−1.32 ± 0.27	84 ± 16	1.59 ± 3.05	16.96 ± 6.77	15.37 ± 4.26	0.90 ± 0.12	2	0.00	0.00	0.00	100.00	0.00	0.00	0.00	0.00	0.00
		A85S		80 ± 13	6.65 ± 4.25	7.85 ± 4.73	1.21 ± 1.56	0.14 ± 0.17	2									
I82S, R107H	2	I82S	0.57 ± 0.74	91 ± 20	38.67 ± 15.10	93.09 ± 13.33	54.42 ± 13.27	0.58 ± 0.12	4	0.00	0.00	0.00	100.00	0.00	0.00	0.00	0.00	0.00
		R107H		123 ± 38	134.74 ± 27.60	197.33 ± 18.99	62.60 ± 21.03	0.32 ± 0.10	2									
I97V, R107C	2	I97V	1.91 ± 0.55	90 ± 19	77.20 ± 19.03	96.61 ± 17.39	19.41 ± 6.87	0.20 ± 0.06	1	0.00	0.00	0.00	0.00	0.00	100.00	0.00	0.00	0.00
		R107C		123 ± 38	134.74 ± 27.60	197.33 ± 18.99	62.60 ± 21.03	0.32 ± 0.10	2									
A104V, I82T	1	A104V	−0.59 ± 0.40	105 ± 27	48.72 ± 14.21	62.41 ± 13.29	13.69 ± 8.89	0.21 ± 0.13	1	0.00	0.00	0.00	0.00	0.00	100.00	0.00	0.00	0.00
		I82T		91 ± 20	38.67 ± 15.10	93.09 ± 13.33	54.42 ± 13.27	0.58 ± 0.12	4									
I82T, V66L	1	I82T	−0.57 ± 0.39	91 ± 20	38.67 ± 15.10	93.09 ± 13.33	54.42 ± 13.27	0.58 ± 0.12	4	0.00	0.00	0.00	0.00	0.00	0.00	100.00	0.00	0.00
		V66L		73 ± 15	14.52 ± 8.56	72.23 ± 9.42	57.72 ± 5.87	0.80 ± 0.07	4									
A85S, I82T	1	A85S	−1.47 ± 0.47	80 ± 13	6.65 ± 4.25	7.85 ± 4.73	1.21 ± 1.56	0.14 ± 0.17	2	0.00	0.00	0.00	0.00	100.00	0.00	0.00	0.00	0.00
		I82T		91 ± 20	38.67 ± 15.10	93.09 ± 13.33	54.42 ± 13.27	0.58 ± 0.12	4									
I82S, V70F	1	I82S	−0.35 ± 0.64	91 ± 20	38.67 ± 15.10	93.09 ± 13.33	54.42 ± 13.27	0.58 ± 0.12	4	0.00	0.00	0.00	100.00	0.00	0.00	0.00	0.00	0.00
		V70F		86 ± 19	15.82 ± 13.93	101.45 ± 11.56	85.63 ± 11.01	0.84 ± 0.10	6									
A69V, I82S	1	A69V	−0.65 ± 0.39	84 ± 16	1.59 ± 3.05	16.96 ± 6.77	15.37 ± 4.26	0.90 ± 0.12	2	0.00	100.00	0.00	0.00	0.00	0.00	0.00	0.00	0.00
		I82S		91 ± 20	38.67 ± 15.10	93.09 ± 13.33	54.42 ± 13.27	0.58 ± 0.12	4									

A104V, V70F	1	A104V	0.09 ± 0.54	105 ± 27	48.72 ± 14.21	62.41 ± 13.29	13.69 ± 8.89	0.21 ± 0.13	1	0.00	0.00	0.00	0.00	0.00	100.00	0.00	0.00	0.00
		V70F		86 ± 19	15.82 ± 13.93	101.45 ± 11.56	85.63 ± 11.01	0.84 ± 0.10	6									
A85V, V70F	1	A85V	0.02 ± 0.47	80 ± 13	6.65 ± 4.25	7.85 ± 4.73	1.21 ± 1.56	0.14 ± 0.17	2	0.00	0.00	0.00	0.00	0.00	0.00	0.00	100.00	0.00
		V70F		86 ± 19	15.82 ± 13.93	101.45 ± 11.56	85.63 ± 11.01	0.84 ± 0.10	6									
A85S, M109V	1	A85S	−0.86 ± 0.40	80 ± 13	6.65 ± 4.25	7.85 ± 4.73	1.21 ± 1.56	0.14 ± 0.17	2	0.00	0.00	0.00	0.00	100.00	0.00	0.00	0.00	0.00
		M109V		118 ± 20	80.28 ± 22.41	168.22 ± 20.18	87.94 ± 14.46	0.52 ± 0.07	5									
A69S, M109L	1	A69S	−1.16 ± 0.30	84 ± 16	1.59 ± 3.05	16.96 ± 6.77	15.37 ± 4.26	0.90 ± 0.12	2	0.00	0.00	0.00	0.00	0.00	100.00	0.00	0.00	0.00
		M109L		118 ± 20	80.28 ± 22.41	168.22 ± 20.18	87.94 ± 14.46	0.52 ± 0.07	5									
A104V, A69S	1	A104V	−1.32 ± 0.28	105 ± 27	48.72 ± 14.21	62.41 ± 13.29	13.69 ± 8.89	0.21 ± 0.13	1	0.00	0.00	0.00	0.00	0.00	100.00	0.00	0.00	0.00
		A69S		84 ± 16	1.59 ± 3.05	16.96 ± 6.77	15.37 ± 4.26	0.90 ± 0.12	2									
A69F, V70L	1	A69F	−0.14 ± 0.36	84 ± 16	1.59 ± 3.05	16.96 ± 6.77	15.37 ± 4.26	0.90 ± 0.12	2	0.00	0.00	0.00	0.00	0.00	0.00	0.00	100.00	0.00
		V70L		86 ± 19	15.82 ± 13.93	101.45 ± 11.56	85.63 ± 11.01	0.84 ± 0.10	6									
A69S, L93M	1	A69S	−1.22 ± 0.23	84 ± 16	1.59 ± 3.05	16.96 ± 6.77	15.37 ± 4.26	0.90 ± 0.12	2	0.00	0.00	0.00	0.00	0.00	0.00	100.00	0.00	0.00
		L93M		80 ± 13	38.00 ± 19.35	107.19 ± 12.26	69.19 ± 9.59	0.65 ± 0.08	4									

Table S3. Equilibration systems details for: i) M protein monomer; systems with M protein and membrane were equilibrated with OPM [1], TMpred [2], TMHMM [3,4], PSIPRED [5,6], CCTOP [7,8] and SACSMEMSAT [9] membrane orientation prediction; ii) M protein dimer, two M monomers with TMHMM [3,4] membrane orientation and membrane were equilibrated. Systems sizes, solvents (water and ions—Na^+ and Cl^-) and membrane lipids (POPC—phosphatidylcholine, Cholesterol, SAPI24 and SAPI25—phosphatidylinositol, POPE—phosphatidylethanolamine, POPS—phosphatidylserine and PSM—sphingolipid) constitution are listed herein.

		Monomer						Dimer
		**OPM**	**TMPRED**	**TMHMM**	**PSIPRED**	**CCTOP**	**SACSMEMSAT**	
**System size**	**x**	14.21	14.21	14.21	14.21	14.21	14.21	11.94
	**y**	14.21	14.21	14.21	14.21	14.21	14.21	11.94
	**z**	12.21	12.21	12.21	12.21	12.21	12.21	11.60
**Solvent**	**H2O**	48949	48918	48954	48935	49005	48935	31319
	**Na^+**	1045	1047	1045	1046	1044	1043	235
	**Cl^-**	797	799	798	798	796	795	83
**Membrane Lipids**	**POPC**	370	370	370	370	370	370	245
	**POPE**	132	132	132	132	132	132	88
	**SAPI24**	30	30	30	30	30	30	26
	**SAPI25**	30	30	30	30	30	30	26
	**POPS**	18	18	18	18	18	18	12
	**PSM**	18	18	18	18	18	18	12
	**Cholesterol**	2	2	2	2	2	2	2

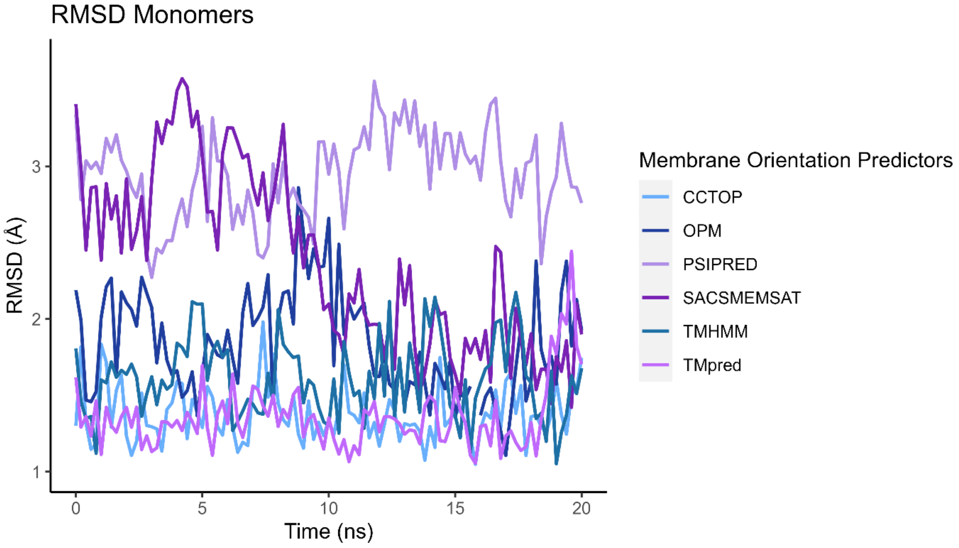

Figure S1. RMSD results for all monomer transmembrane predictions for SARS-CoV-2 M protein during the equilibration.

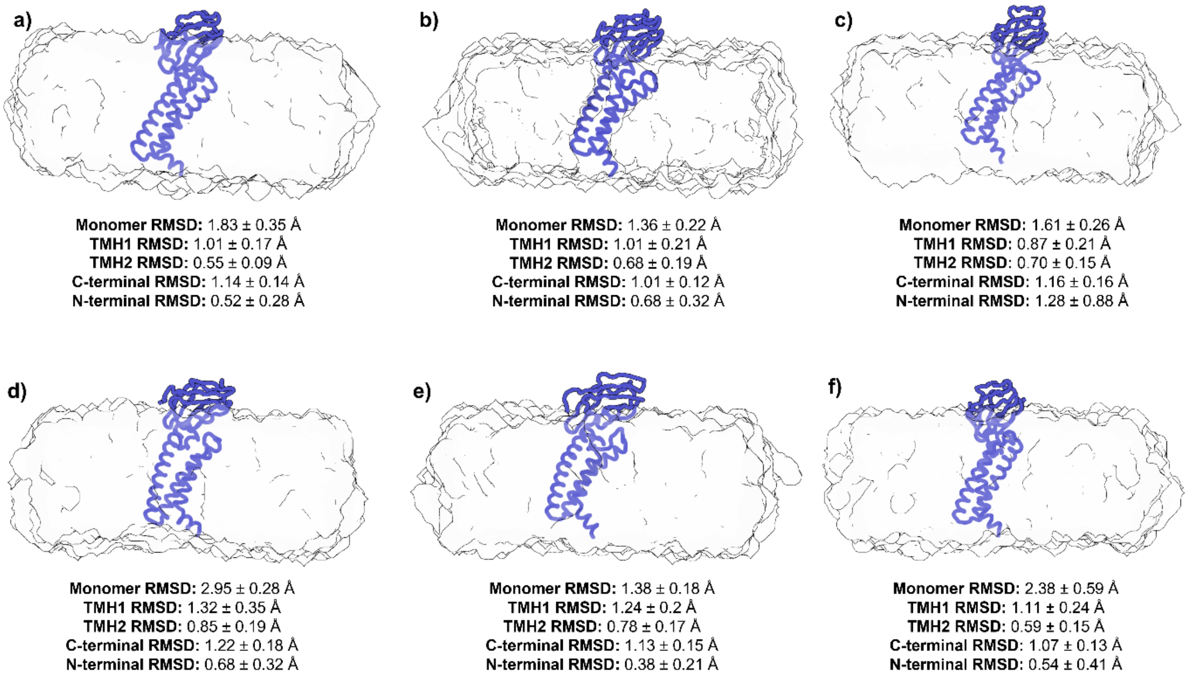

Figure S2. SARS-CoV-2 M protein monomer transmembrane prediction: OPM [1], TMpred [2], TMHMM [3,4], PSIPRED [5,6], CCTOP [7,8] and SACSMEMSAT [9].

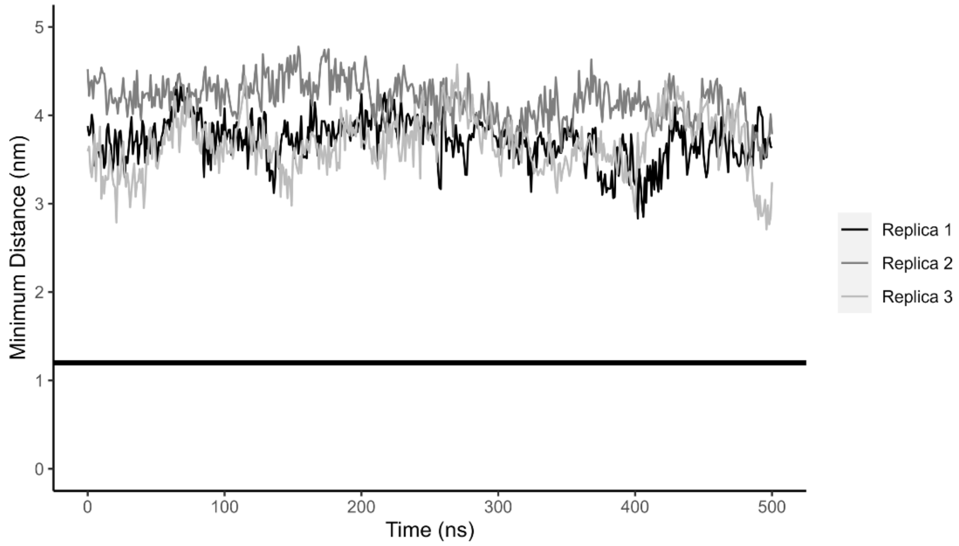

Figure S3. Minimum Distance between periodic imagens during the MD simulation for the three replicas. Straight horizontal black line, at value 1.2, represents the minimal acceptable distance between periodic images to avoid direct interactions between neighboring images. All replicas showed a minimum distance superior to the threshold.

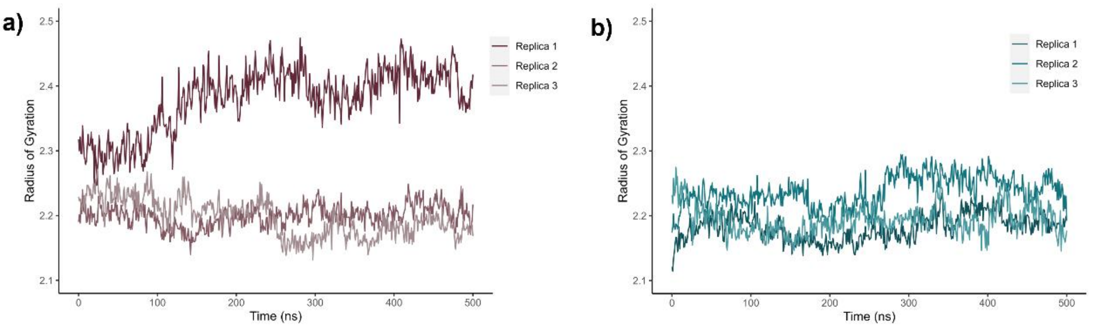

Figure S4. Radius of gyration of (**a**) monomer A and (**b**) monomer B three replicas throughout the MD simulation time. Monomer A shows a stable radius of gyration. However, the values for monomer B only became stable after 300 ns, which justifies the use of just the last 200 ns for subsequent analysis.

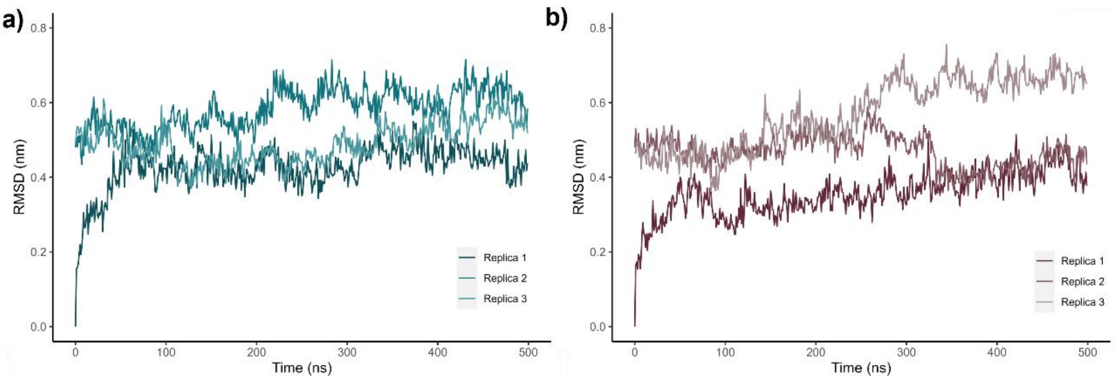

Figure S5. RMSD values of (**a**) monomer A and (**b**) monomer B three replicas throughout the MD simulation time. RMSD values became more stable after 300 ns, further strengthening the decision to use the last 200 ns for subsequent analysis.

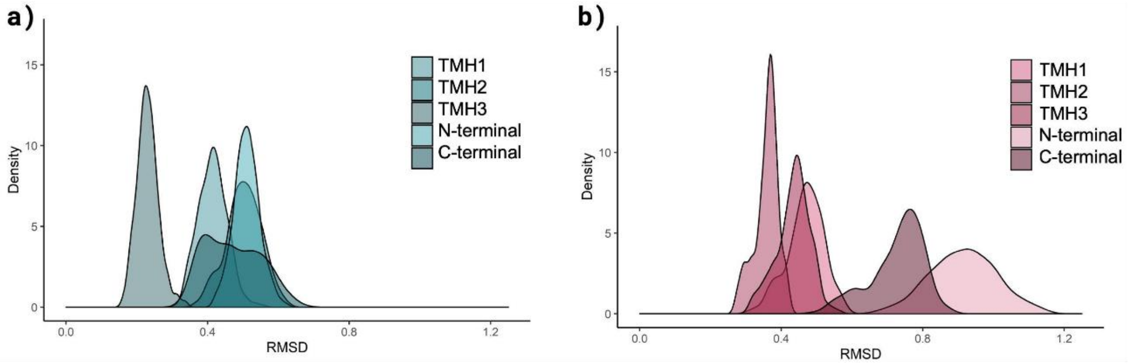

Figure S6. RMSD results for MD simulations split by TMH1, TMH2, TMH3, N-terminal and C-terminal: (**a**) monomer A, and (**b**) monomer B.

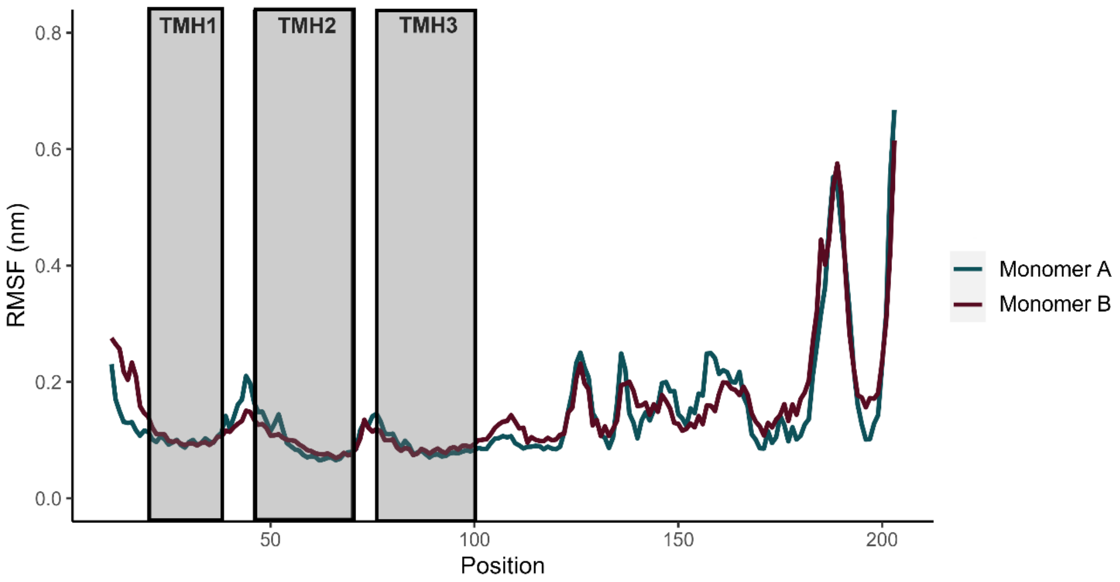

Figure S7. RMSF results for MD simulations split by monomers A and B. TMH1, TMH2 and TMH3 correspondent residues are highlighted as grey areas.

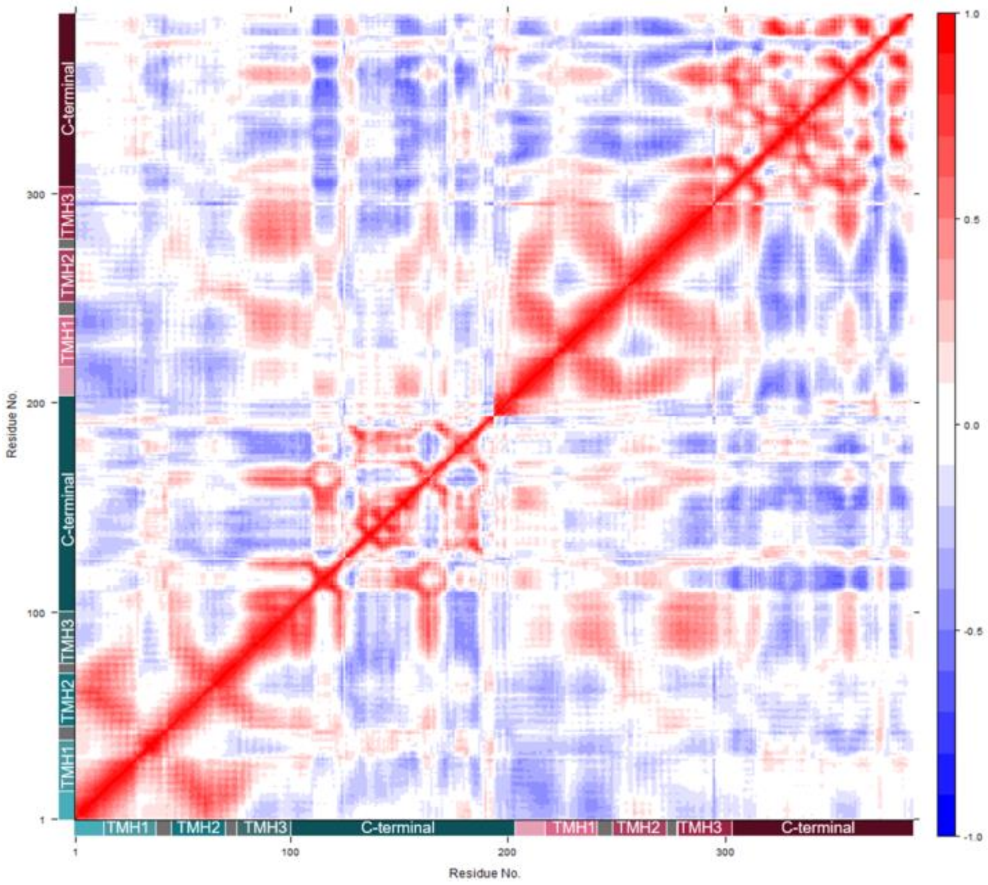

Figure S8. Residue CCA between M protein dimer monomer A and monomer B. All regions are colored according to Figure 3 color scheme.

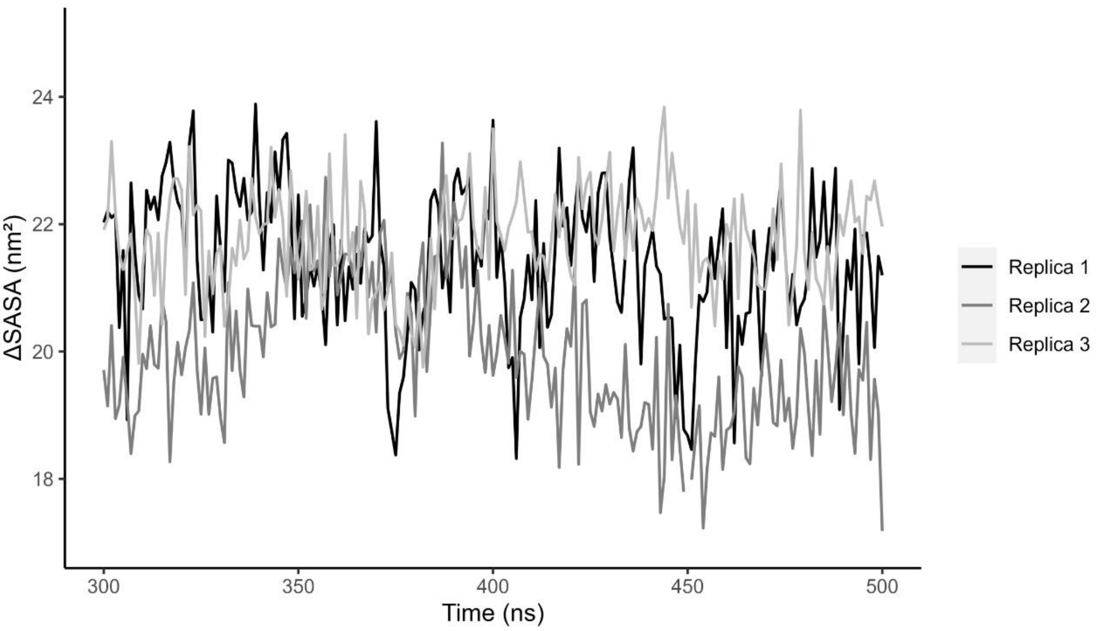

Figure S9. Average ΔSASA values of the interfacial residues. ΔSASA values were calculated by subtracting the SASA values of the complex with the SASA values of the individual monomers. This plot clearly shows the constancy of this values across the simulations, further demonstrating the convergence of the simulations.

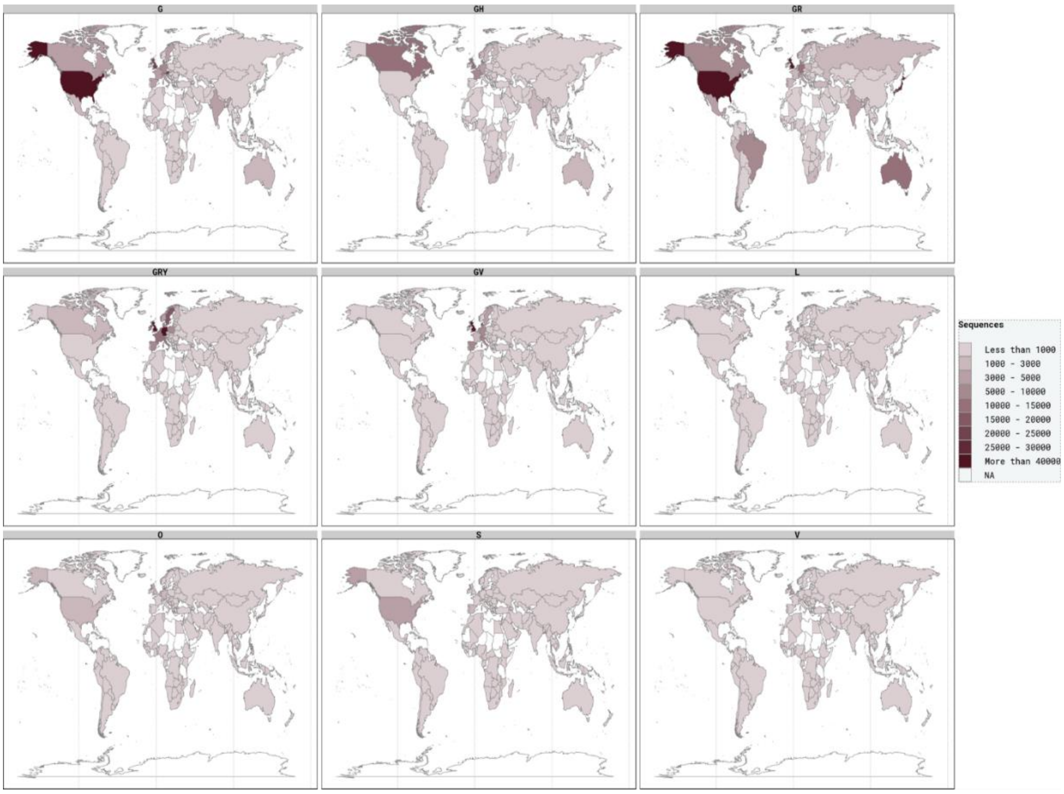

Figure S10. GISAID data analysis by clades. Clade S includes variants A, clade V variants B.2, clade L variants B, clade G variants B.1, clade GH variants B.1.*, clade GV variants B.1.177, clade GR variants B.1.1.1 and clade GRY variants B.1.1.7.

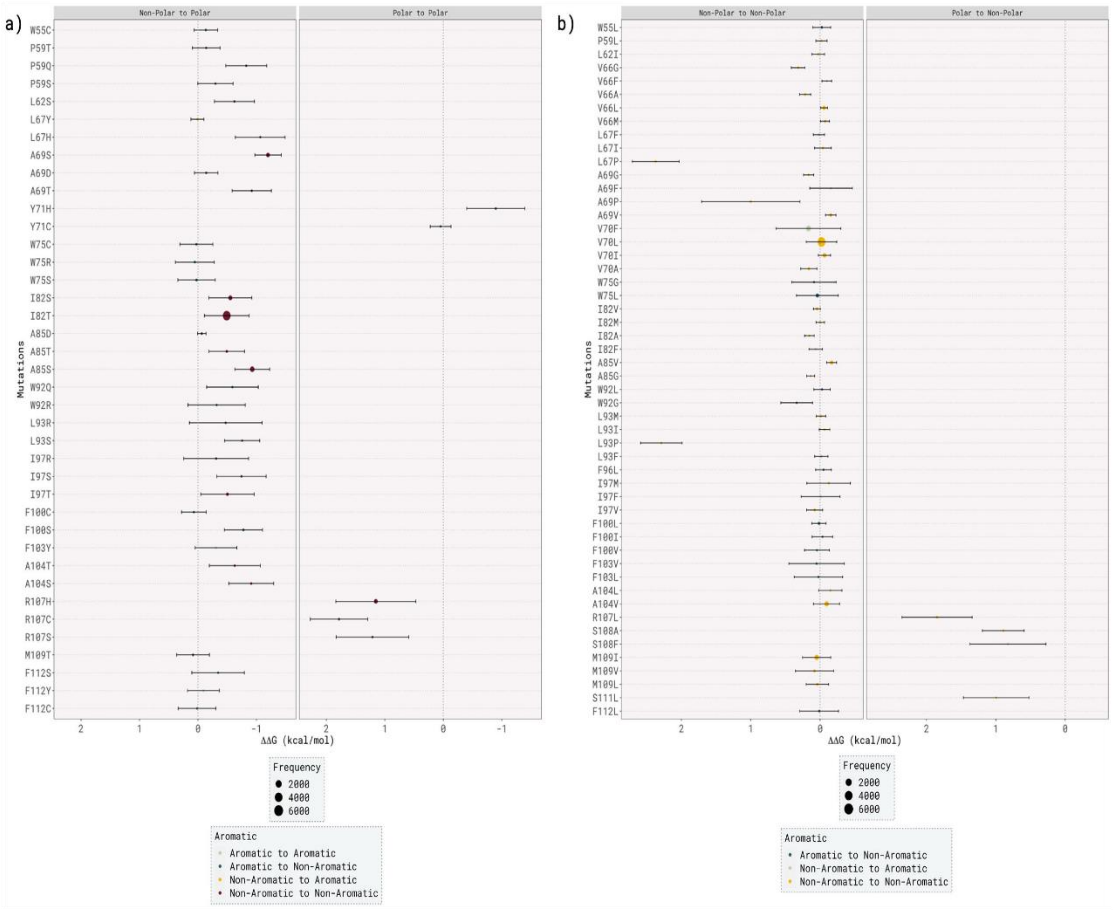

Figure S11. $\triangle\triangle G_{binding}$ values of predicted interfacial residues split into (**a**) Non-polar to Non-polar residues and Polar to Non-polar residues; and (**b**) Non-Polar to Polar residues and Polar to Polar residues. Size corresponds to the number of times a SNP occurred in all 1,271,550 analyzed sequences. Color represents the alteration from Aromatic to Aromatic (sage), Aromatic to Non-aromatic (teal), Non-aromatic to Aromatic (yellow) and Non-aromatic to Non-aromatic (garnet) (all the presented results are mean values ± standard deviation).

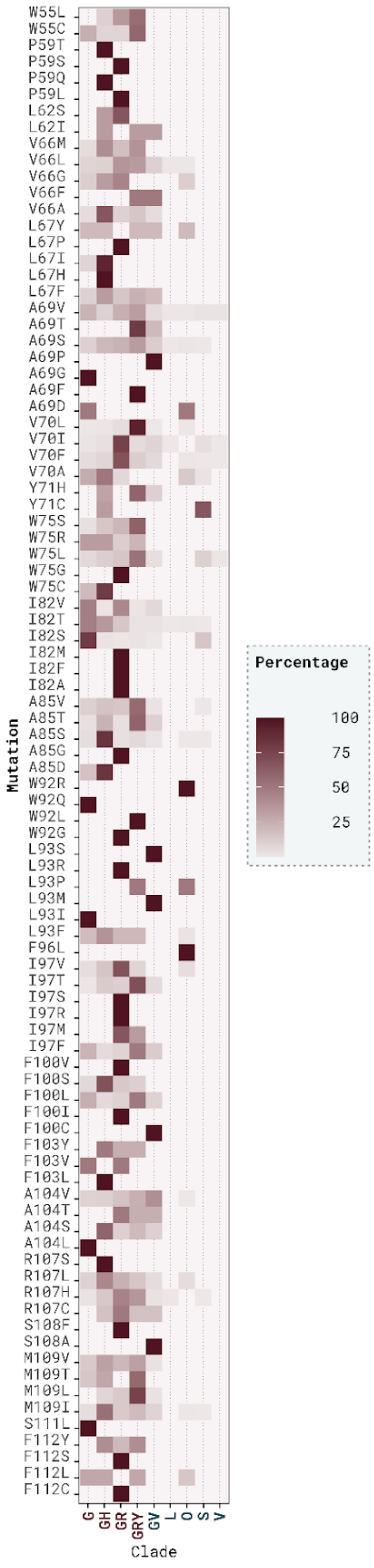

Figure S12. Distribution across Clades of SARS-CoV-2 M protein sequences. Clade color is related to it encompassing VOC (garnet) and VOI (teal).

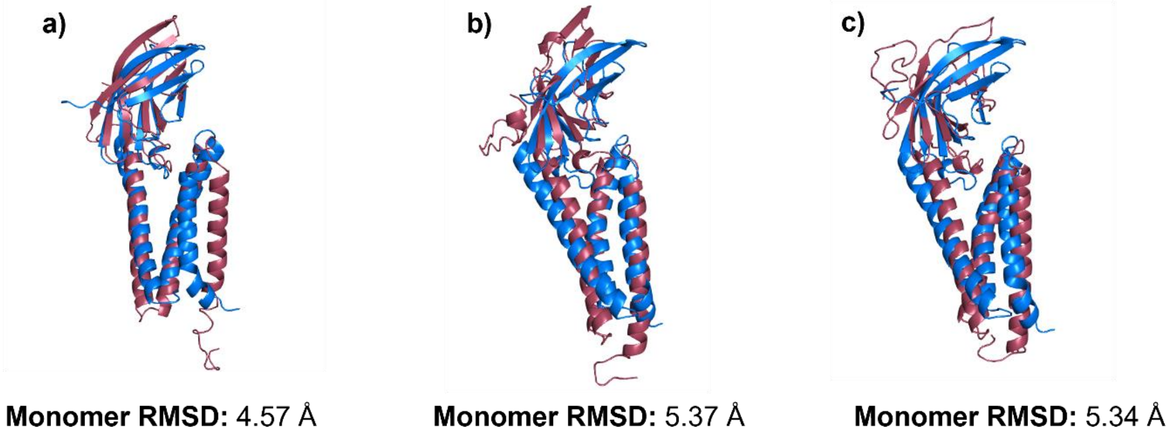

Figure S13. Overlay of the obtained monomer after equilibration (blue) and: (**a**) the Feig's laboratory "second" monomer prediction [10]; (**b**) The Feig's laboratory "first" monomer prediction [10]; (**c**) Zhang et al. monomer prediction [11]. Mean values for the RMSD between both structures are also shown.

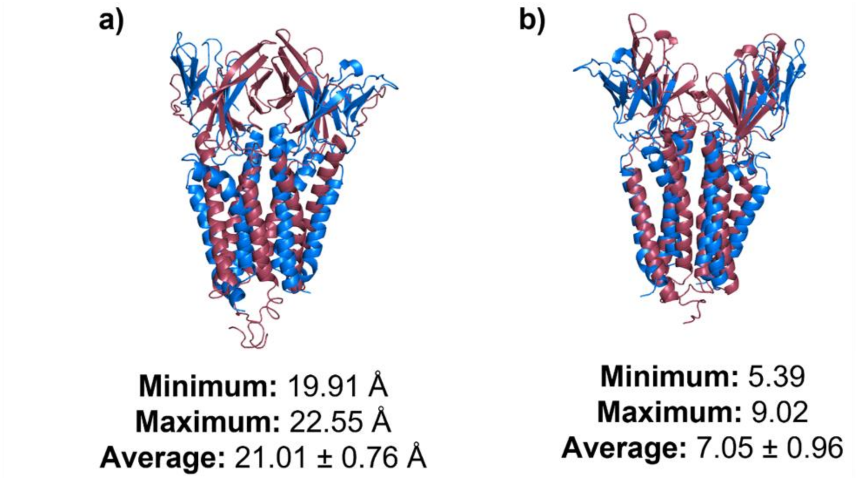

Figure S14. Overlay of the predicted homodimer structure (blue) and (**a**) the most recent homodimer predicted by Feig's laboratory ("closed" conformation) [12]; (**b**) the first homodimer predicted by Feig's laboratory ("open conformation") [12]. Mean and standard deviation values for the RMSD between both structures are also shown.

References

1. Lomize, M.A.; Pogozheva, I.D.; Joo, H.; Mosberg, H.I.; Lomize, A.L. OPM database and PPM web server: Resources for positioning of proteins in membranes. *Nucleic Acids Res.* **2012**, *40*, D370–D376.
2. Hofman, K. TMbase-a database of membrane spanning proteins segments. *Biol. Chem. Hoppe Seyler.* **1993**, *374*, 166.
3. Sonnhammer, E.L.; von Heijne, G.; Krogh, A. A hidden Markov model for predicting transmembrane helices in protein sequences. Proc. Int. Conf. Intell. Syst. Mol. Biol. 1998, 6, 175–182
4. Krogh, A.; Larsson, B.; von Heijne, G.; Sonnhammer, E.L. Predicting transmembrane protein topology with a hidden Markov model: Application to complete genomes. *J. Mol. Biol.* **2001**, *305*, 567–580.
5. Buchan, D.W.A.; Jones, D.T. The PSIPRED Protein Analysis Workbench: 20 years on. *Nucleic Acids Res*. **2019**, *47*, W402–W407.
6. Jones, D.T. Protein secondary structure prediction based on position-specific scoring matrices. *J. Mol. Biol*. **1999**, *292*, 195–202.
7. Dobson, L.; Reményi, I.; Tusnády, G.E. CCTOP: A Consensus Constrained TOPology prediction web server. *Nucleic Acids Res.* **2015**, *43*, W408–W412.
8. Dobson, L.; Reményi, I.; Tusnády, G.E. The human transmembrane proteome. *Biol. Direct.* **2015**, *10*, 31.
9. Jones, D.T.; Taylor, W.R.; Thornton, J.M. A model recognition approach to the prediction of all-helical membrane protein structure and topology. *Biochemistry* **1994**, *33*, 3038–3049.
10. Heo, L.; Feig, M. Modeling of severe acute respiratory syndrome coronavirus 2 (SARS-CoV-2) proteins by machine learning and physics-based refinement. *bioRxiv.* **2020**, doi:10.1101/2020.03.25.008904.

11. Zhang, C.; Zheng, W.; Huang, X.; Bell, E.W.; Zhou, X.; Zhang, Y. Protein structure and sequence reanalysis of 2019-nCoV genome refutes snakes as its intermediate host and the unique similarity between its spike protein insertions and HIV-1. *J. Proteome Res.* **2020**, *19*, 1351–1360.
12. Feig, M. SARS-Cov-2 Protein Structure Models. Available online: https://github.com/feiglab/sars-cov-2-proteins (accessed on 19 February 2022).
